# Exosomal transfer of stroma-derived miR21 confers paclitaxel resistance in ovarian cancer cells through targeting APAF1

**DOI:** 10.1038/ncomms11150

**Published:** 2016-03-29

**Authors:** Chi Lam Au Yeung, Ngai-Na Co, Tetsushi Tsuruga, Tsz-Lun Yeung, Suet-Ying Kwan, Cecilia S. Leung, Yong Li, Edward S. Lu, Kenny Kwan, Kwong-Kwok Wong, Rosemarie Schmandt, Karen H. Lu, Samuel C. Mok

**Affiliations:** 1 Department of Gynecologic Oncology and Reproductive Medicine, The University of Texas MD Anderson Cancer Center, 1515 Holcombe Boulevard, Unit 1362, Houston, Texas 77030, USA; 2 Department of Biochemistry and Molecular Biology, University of Louisville, Louisville, Kentucky 40202, USA

## Abstract

Advanced ovarian cancer usually spreads to the visceral adipose tissue of the omentum. However, the omental stromal cell-derived molecular determinants that modulate ovarian cancer growth have not been characterized. Here, using next-generation sequencing technology, we identify significantly higher levels of microRNA-21 (miR21) isomiRNAs in exosomes and tissue lysates isolated from cancer-associated adipocytes (CAAs) and fibroblasts (CAFs) than in those from ovarian cancer cells. Functional studies reveal that miR21 is transferred from CAAs or CAFs to the cancer cells, where it suppresses ovarian cancer apoptosis and confers chemoresistance by binding to its direct novel target, APAF1. These data suggest that the malignant phenotype of metastatic ovarian cancer cells can be altered by miR21 delivered by exosomes derived from neighbouring stromal cells in the omental tumour microenvironment, and that inhibiting the transfer of stromal-derived miR21 is an alternative modality in the treatment of metastatic and recurrent ovarian cancer.

## Abstract (web summary)

The tumor microenviroment can influence cancer progression and response to therapy. In this study, the authors show that miR21 is transferred through exosomes from cancer-associated fibroblasts and adipocytes to ovarian cancer cells where it modulates drug resistance by its direct target APAF1.

## Graphical abstract

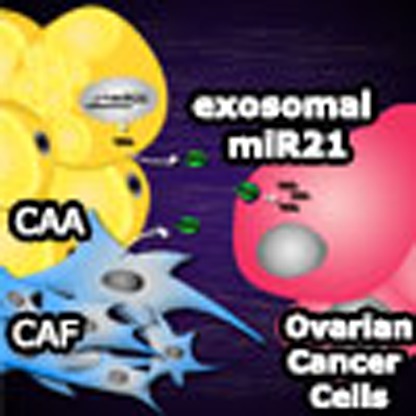

Approximately 22,000 new cases of epithelial ovarian cancer have been diagnosed in the United States in 2015 (ref. 1). Over 16,000 deaths per year occurred, making this cancer the most lethal gynaecologic malignancy. Although cancer patients initially respond to platinum- and taxane-based chemotherapy following surgery, most of them experience recurrence within 12–24 months and die of progressively chemotherapy-resistant disease.

One critically important, yet often overlooked, component of tumour progression is the tumour microenvironment, which is primarily composed of fibroblasts, extracellular matrix proteins, endothelial cells and lymphocytic infiltrate. The tumour microenvironment has been shown to directly affect cell growth, migration and differentiation through secreted proteins, cell–cell interactions and matrix remodelling^2^. As it can promote the tumour initiation of normal epithelial cells and facilitate the progression of malignant cells, the tumour microenvironment presents a unique opportunity to discover ways to better diagnose, understand and treat cancer. Recent studies have shown that in addition to initiation via soluble mediators, cell–cell communication can be initiated via surface interactions between circulating exosomes and transmembrane molecules expressed by target cells^3^. The fusion of exosomes with target cell membranes facilitates the transfer of cell surface molecules and receptors from donor to recipient cells^3^. Furthermore, the endocytosis of exosomes by their target cells results in the intracellular release of vesicular contents, including messenger RNA, microRNA (miRNA), proteins and lipids^4^. Tumour exosomes have been shown to have angiogenic properties. For example, colorectal cancer exosomes transfer mRNAs, which promote endothelial cell proliferation and facilitate angiogenesis^5^, whereas glioblastoma-derived exosomes promote tubule formation by recipient endothelial cells^6^. Furthermore, tumour exosomes secrete factors that suppress natural killer cell activity and induce T-cell apoptosis^7^. In this way, the tumour cells themselves create a ‘tumour-friendly' environment that promotes cancer metastasis and progression.

The transfer of miRNA by exosomes is particularly interesting, because miRNAs are more stable and can control the expression of multiple target genes in the recipient cells. In addition, miRNAs have been shown to regulate cell differentiation, proliferation and apoptosis, and contribute to the development of multiple tumour types^8^,^9^,^10^,^11^. Although the miRNA signatures of tumour-derived exosomes have been identified in multiple tumour types, including ovarian cancer^12^,^13^, exosomal miRNA signatures from cancer-associated stromal cells have not been investigated and the functional roles of these exosomal miRNAs in modulating the malignant phenotypes of recipient cancer cells have not been elucidated.

In this study, we use next-generation sequencing to identify differential miRNA signatures in exosomes isolated from ovarian cancer cells and ovarian cancer-associated fibroblasts (CAFs) and adipocytes (CAAs). We demonstrate that specific miRNAs are directly transferred, through exosomes, from CAFs and CAAs to ovarian cancer cells, and we identify the molecular mechanisms by which miRNAs modulate the malignant phenotypes in ovarian cancer cells.

## Results

### CAF and CAA exosomes have higher miR21 copy number

miRNAs that transfer between living cells that are involved in cell–cell communication are frequently encapsulated in exosomes, which facilitate their targeted exchange^14^. To identify miRNAs that are transferred by exosomes secreted from omental stromal cells to ovarian cancer cells, we generated miRNA profiles of CAFs, CAAs and ovarian cancer cells by Ion Torrent next-generation sequencing (GEO #GES77318). Primary CAFs and CAAs were first isolated from ovarian tumour tissue samples and characterized by α-smooth muscle actin staining and adiponectin mRNA expression, respectively. The α-smooth muscle actin expression level in CAFs that we used was significantly higher than that in the normal ovary fibroblasts (Supplementary Fig. 1a). The difference in expression was consistent with that documented in other cancer types^15^,^16^. The adiponectin expression levels in the CAAs that we used were significantly lower than those in normal adipocytes (Supplementary Fig. 1b). Similar findings have been reported in adipocytes associated with several cancer types such as gastric cancer^17^ and prostate cancer^18^.

**Figure 1:**
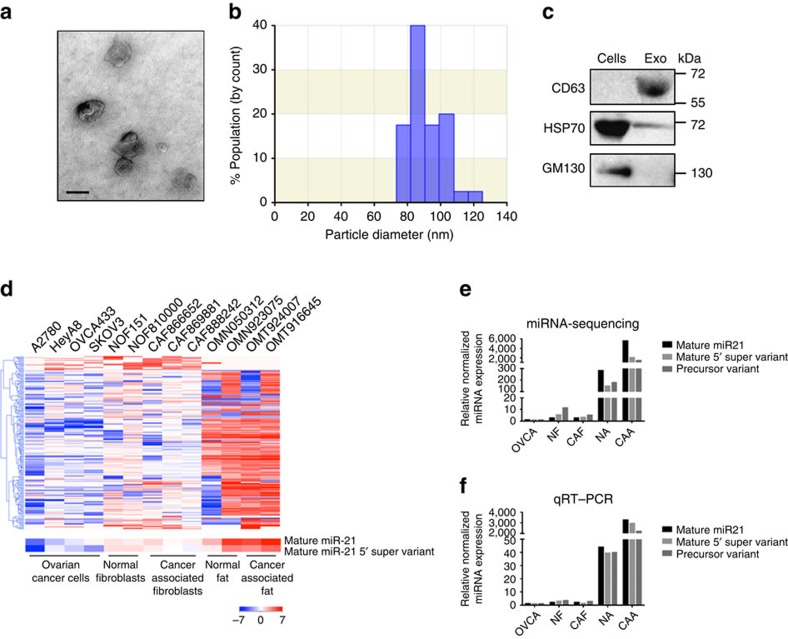
Increased miR21 expression in exosomes isolated from CAAs. (**a**) Electron micrograph showing whole-mount exosomes isolated from CAA-conditioned medium. Scale bar, 100 nm. (**b**) Histogram showing the particle diameter (nm) of the population of small vesicles collected from CAA-conditioned medium using the standard exosome isolation method of ultracentrifugation and quantified using qNano. The amount of exosomes secreted into the conditioned media was normalized with the number of cells in the culture for conditioned media collection. The amount of miR-21 in the exosomes was then normalized with the input amount of RNA for reverse transcription and the subsequent qRT–PCR analysis, to determine the relative miR-21 expression level in exosomes. (**c**) Western blot analysis showing exosome marker CD63 in the exosome-enriched conditioned medium but not in the cell lysate. In contrast, *cis*-Golgi matrix protein (GM130) was only observed in the cell lysate and not in the exosome fraction. Another exosome marker, 70-kDa heat shock protein (HSP70), was also observed in the exosomes. (**d**) The heat map showing the relative expression of small RNAs in exosomes isolated from ovarian cancer cell lines (*n*=4), normal ovarian fibroblasts (*n*=2), CAFs (*n*=3), normal adipocytes (*n*=2) and CAAs (*n*=2). (**e**,**f**) Relative normalized expression of mature 5′, mature 5′ super variant and precursor variant of miR21 in exosomes isolated from ovarian cancer cell lines (*n*=4), normal ovarian fibroblasts (*n*=2), CAFs (*n*=3), normal adipocytes (*n*=2) and CAAs (*n*=2) examined using miRNA-sequencing (**e**) and qRT–PCR (**f**) analyses.

Once we characterized the cultured cells that we obtained, we isolated the exosomes from the conditioned media collected from each cell type. Exosomes were first characterized and quantified by electron microscopy, qNano analysis and western blot analysis. The double-layer membrane nature and size of the exosomes were confirmed by electron microscopy and qNano analysis (Fig. 1a,b). A western blot analysis showed the expression of the exosomal markers CD63 and HSP70 but the absence of the *cis*-Golgi compartment-specific marker GM130 (Fig. 1c). A qNano analysis of media collected from an equal number of cells also demonstrated that a higher number of exosomes was generated from CAFs than from CAAs and ovarian SKOV3 cancer cells (Supplementary Table 1).

Next-generation sequencing was performed on RNA samples extracted from exosomes collected from ovarian cancer cell lines, normal ovarian fibroblasts lines, ovarian CAF lines established from primary tumours, CAAs isolated from omental tumours and normal omental adipocytes isolated from omental tissue in patients with benign gynaecologic diseases. The number of sequencing reads for each miRNA and their variants was normalized by adjusting the counts per 1,000 cells (Fig. 1d and Supplementary Data 1). Data analyses identified a diverse population of variants of miRNAs that are collectively known as isomiRNAs (isomiRs), which are miRNAs that have variations in the sequences with respect to the reference miRNA sequence. The variations include 3′-trimming, 5′-trimming (collectively as sub), 3′-nucleotide addition (super) and nucleotide substitution (variant). The variations of the sequences are mostly non-random and evolutionarily conserved 3′-nucleotide additions or modifications; other isomiRs appear to be derived from variability in Dicer 1 and Drosha cleavage sites. As isomiRs share most of their sequence with the most highly expressed form of miRNAs (usually the miRNAs with the conventional sequence), it is possible that these variants share a common set of targets^19^.

A supervised clustering analysis showed distinct miRNA transcriptome signatures among ovarian cancer cells, fibroblasts and adipocytes (Fig. 1d). Among the miRNAs and isomiRs that were identified in exosomes, miR21 and its variants were the most abundant. Among the 84 different forms of miR-21 isomiR that we identified in exosomes, 8 (including the conventional mature 5′-miR-21 listed in the miRbase) were expressed in all the cell types that we examined. The mature 5′ super variant isomiR (5′-TAGCTTATCAGACTGATGTTGACA-3′) and the mature 5′ conventional miR21 (5′-TAGCTTATCAGACTGATGTTGA-3′) were the most abundant in exosomes isolated from CAFs and CAAs. The expression of mature 5′-miR21 was second highest in exosomes derived from CAFs and CAAs following the mature 5′ super variant, which had the highest copy number in the exosomes isolated from both CAFs and CAAs. They also had higher copy numbers in normal ovarian fibroblasts, CAFs, normal adipocytes and CAAs than in cancer cells, with CAAs demonstrating the highest copy numbers (Table 1 and Supplementary Data 2), indicating that CAAs produced the highest numbers of exosomal miR21 isomiRs.

The expression levels of the two isomiRs with the highest levels in CAFs and CAAs (mature 5′ super variant and precursor variant: 5′-TAGCTTATCAGACTGATGTTGACTA-3′), together with that of the mature 5′ conventional miR21, were validated by quantitative reverse transcriptase–PCR (qRT–PCR) analysis. The results showed high concordance between the miRNA-seq and qRT–PCR data. All three miR21 variants showed higher expression levels in the four stromal cell types compared with those in ovarian cancer cells. The mature 5′-miR21 had the largest fold difference in expression levels between CAAs and ovarian cancer cells, whereas the precursor variant had the largest fold difference between CAFs and ovarian cancer cells (Fig. 1e,f and Supplementary Table 2).

### Different levels of miR21 in stromal and tumour tissues

**Figure 2:**
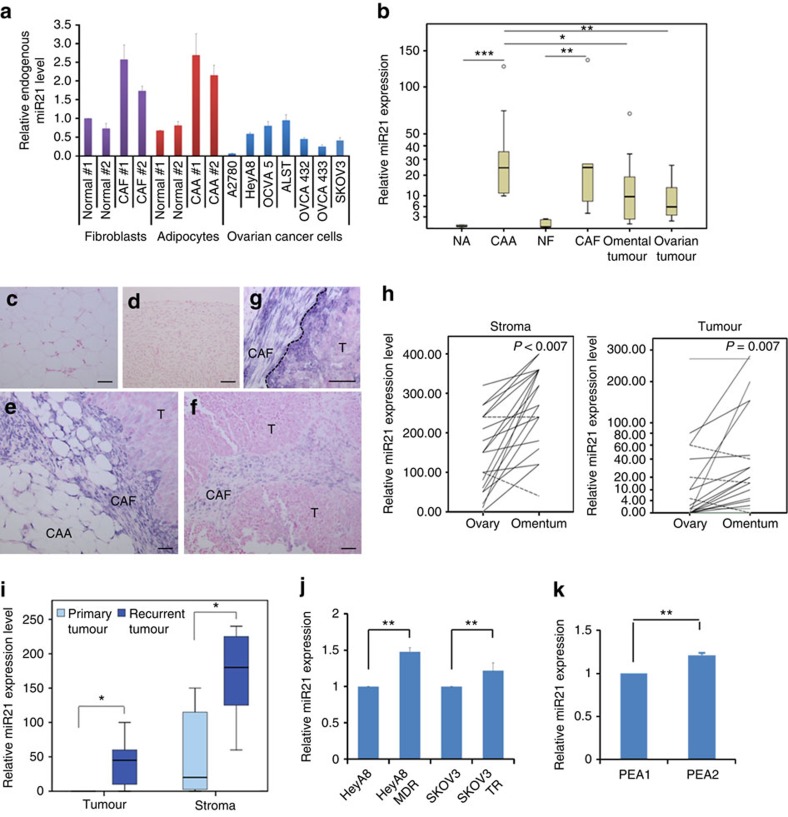
Differential miR21 expression in ovarian tumour tissue and cancer cell lines. (**a**) qRT–PCR analysis of miR21 expression in cultured normal ovarian fibroblasts, CAFs, normal adipocytes, CAAs and ovarian cancer cell lines. (**b**) qRT–PCR analysis of miR21 expression in microdissected normal adipocytes (*n*=5), CAAs (*n*=9), normal fibroblasts (*n*=5), CAFs (*n*=5) and ovarian cancer cells from omental (*n*=10) and primary ovarian sites (*n*=10). ***P<0.001, **P<0.01 and *P<0.05; Mann–Whitney *U*-test. (**c**–**g**) *In situ* hybridization of miR21 in (**c**) normal omental adipocytes, (**d**) normal ovarian tissue and (**e**) omental and (**f**) primary ovarian sites of ovarian carcinoma. Strong miR21 staining in omental cancer cells was observed at the tumour–stroma interface (**g**; dashed line). Digoxigenin-labelled miR21 probes were detected using anti-digoxigenin-alkaline phosphatase and visualized as dark blue nitro-blue tetrazolium and 5-bromo-4-chloro-3′-indolyphosphate precipitate. Sections were counterstained with Nuclear Fast Red. CAAs, cancer-associated adipocytes; CAFs, cancer-associated fibroblasts; T, tumour. Scale bar, 50 μm. (**h**) Pair-wise comparisons of miR21 expression in omental and primary ovarian tumour sites from stromal (*n*=20; *P*<0.000; Wilcoxon signed-rank test) and epithelial (*n*=19; *P*=0.007; Wilcoxon signed-rank test) components of advanced-stage ovarian cancer cells. Relative miR21 expression was measured by *in situ* hybridization; each line represents the expression level in the same patient. The solid lines indicate higher miR21 expression in the omental site than in the primary ovarian site; the dashed lines represent lower expression in the omental site. (**i**) Box plot showing miR21 expression in stromal (*n*=14) and epithelial (*n*=14) components of primary and recurrent omental ovarian tumour tissue, as quantified by *in situ* hybridization analyses. **P<0.01; Mann–Whitney *U*-test. (**j**) qRT–PCR of miR21 expression in ovarian cancer HeyA8 and SKOV3 cells and their paclitaxel-resistant sublines HeyA8-MDR and SKOV3-TR. The results were the average from at least three separate experiments. Mean±s.d.; **P<0.01; two-tailed Student's *t*-test. (**k**) qRT–PCR of miR21 expression in the primary PEA1 tumour cells and the cell line established after cisplatin treatment (PEA2). The results were the average from at least three separate experiments. Mean±s.d.; **P<0.01; two-tailed Student's *t*-test.

To determine whether the higher mature 5′ conventional miR21 copy numbers identified in CAA- and CAF-derived exosomes were due to significantly higher miR21 expression in CAAs and CAFs than in ovarian cancer cells, we performed a qRT–PCR analysis on RNA isolated from primary normal adipocytes (NAs), CAAs, normal fibroblasts (NFs), CAFs and ovarian cancer cells. The results showed that both CAAs and CAFs expressed significantly higher levels of endogenous miR21 than did ovarian cancer cells and normal stromal cells (Fig. 2a). To further confirm the differential miR21 expression in the stromal and epithelial components of ovarian tumour tissue, we performed qRT–PCR analyses on microdissected normal adipocytes isolated from normal omental tissue, CAAs, normal ovarian fibroblasts, CAFs and ovarian cancer cells derived from primary ovarian and omental sites. The results showed that CAAs expressed the highest levels of miR21 (Fig. 2b). In addition, both normal adipocytes and normal ovarian fibroblasts expressed the lowest levels of miR21 and omental ovarian tumours expressed higher levels of miR21 than did those in primary ovarian tumours (Fig. 2b).

To further evaluate the differential expression patterns of miR21 in different components of normal and malignant ovarian tissue, we performed *in situ* hybridization on an independent set of tissue samples. The results confirmed that normal omental adipocytes and fibroblasts expressed the lowest levels of miR21 (Fig. 2c,d). In addition, the highest levels of miR21 observed in the tumour cells were in the stromal–epithelial interface and the invasion front (Fig. 2e–g), suggesting that miR21 is either upregulated by mediators secreted from neighbouring stromal cells or directly transferred from neighbouring cells. A pairwise comparison demonstrated that in both the tumour and stroma, the cancer in the omental site expressed significantly higher levels of miR21 than did the cancer in the primary site in the same patient (*P*<0.001 and *P*=0.007, respectively) (Fig. 2h). Moreover, recurrent ovarian cancer showed significantly higher levels of miR21 expression than did primary ovarian cancer in both the stromal and the epithelial components of ovarian cancer tissue (Fig. 2i). *In vitro*, increased miR21 expression levels were observed in the chemoresistant cell lines HeyA8 MDR and SKOV3TR compared with those in their parental lines HeyA8 and SKOV3, respectively (*P*<0.01) (Fig. 2j). Furthermore, a higher miR21 expression level was observed in the cell line PEA2, established from the recurrent tumour of a pretreated ovarian cancer patient than in PEA1 primary tumour cells from the same patient (*P*<0.01) (Fig. 2k). The increase in miR21 expression levels in recurrent or chemoresistant ovarian tumour cells was much lower *in vitro* (<1.5-fold) than *in vivo* (>10-fold), suggesting that the stromal cells in the tumour microenvironment increase miR21 expression levels in ovarian cancer cells through the direct transfer of miR21 or transcriptionally by soluble mediators.

### Direct transfer of miR21 from CAF and CAA to cancer cells

**Figure 3:**
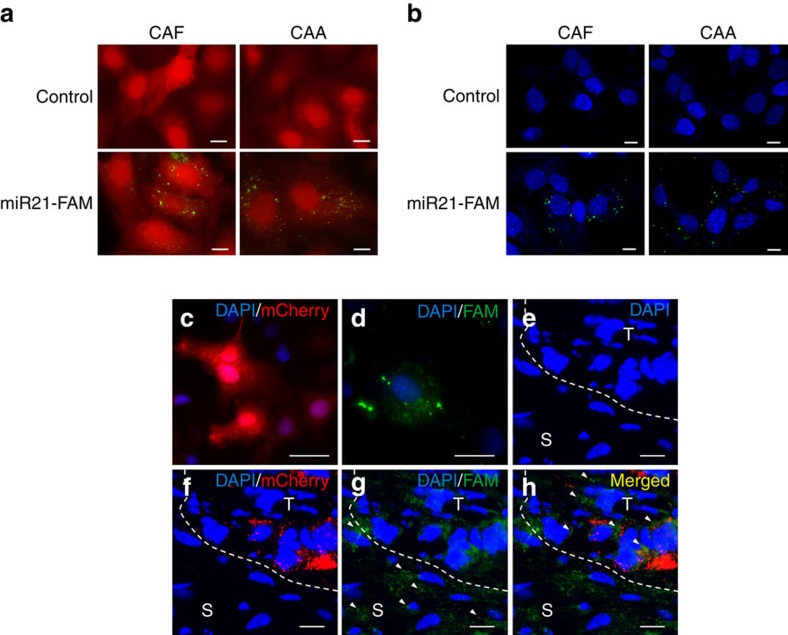
Exosomal transfer of miR21 from adipocytes and fibroblasts to ovarian cancer cells. (**a**) CAAs and CAFs transiently transfected with FAM-tagged miR21 (miR21-FAM) or without transfection (Control) were co-cultured with mCherry-labelled ovarian cancer SKOV3ip cells for 24 h. Fluorescence microscopy was used to detect the green and red fluorescent signals in SKOV3ip cells. Scale bar, 2 μm. (**b**) Exosomes were isolated from conditioned media prepared from CAAs and CAFs transfected with FAM-labelled miR21 (miR21-FAM) or without transfection (Control) and added to ovarian cancer SKOV3ip cell cultures. SKOV3ip cells were fixed and the nuclei were stained with 4,6-diamidino-2-phenylindole (DAPI) blue. An SP5 confocal microscope was used to detect the green signals in SKOV3ip cells. Scale bar, 2 μm. (**c**–**h**) Red mCherry-labelled ovarian cancer SKOV3ip cells (**c**) were injected subcutaneously into nude mice, to establish tumours. After 1 week, ^miR21−/miR21−^MEFs transfected with FAM-tagged miR21 (**d**) were injected intratumorally. After 24 h, tumours were harvested and frozen sections were prepared. A confocal microscopy analysis showed the blue DAPI signals for the nuclei of the two cell types (**e**), the red mCherry signals for the SKOV3ip cells (**f**) and the green FAM signals for miR21 (**g**). Arrowheads indicate the FAM-miR21 signals in the peripheral stromal cells (S) and in some of the red cancer cells (T) (**g**,**h**). Scale bar, 5 μm.

To determine whether miR21 can be directly transferred from CAAs or CAFs to ovarian cancer cells, we co-cultured ovarian cancer SKOV3ip cells, which have a comparable miR21 expression level as SKOV3 cells, with CAFs and CAAs that were transiently transfected with fluorescein amidite (FAM)-tagged miR21. Confocal microscopy detected fluorescently labelled miR21 in the cancer cells co-cultured with CAFs and CAAs but not in those co-cultured with mock-transfected CAAs and CAFs, suggesting that miR21 was transferred from the CAAs and CAFs to the cancer cells (Fig. 3a). To further delineate whether the transfer of miR21 is mediated by exosomes, we first isolated exosomes from conditioned media collected from CAFs and CAAs that had been transiently transfected with FAM-tagged miR21 or the pre-miR negative control. The purified exosomes were then added to SKOV3ip cells. Confocal microscopy detected fluorescently labelled signals in the cancer cells incubated with exosomes obtained from FAM-tagged miR21-transfected cells but not in those incubated with exosomes from mock-transfected cells (Fig. 3b), confirming that miR21 was transferred from CAAs and CAFs to ovarian cancer cells via exosomes.

Next, we determined whether CAA-derived exosomes, which contained higher levels of miR21 than did those from CAFs, transferred more miR21 to recipient cells. Ovarian cancer SKOV3 cells were treated with the same number of exosomes isolated from CAAs and CAFs. A qRT–PCR analysis of miR21 was performed of the recipient cells. The results showed that miR21 expression levels were higher in SKOV3 cells treated with exosomes isolated from CAAs than in those treated with exosomes from CAFs (Supplementary Fig. 2a). These data suggest that the amount of miR21 transferred to the recipient cells depends on the amount of miR21 delivered in the exosomes.

To determine whether CAF-derived miR21 can be transferred to ovarian cancer cells *in vivo*, we injected red mCherry-labelled ovarian cancer SKOV3ip cells (Fig. 3c) subcutaneously into nude mice. Once the tumour nodules had formed^19^, ^miR21−/miR21−^mouse embryonic fibroblasts (^miR21−/miR21−^MEFs) were transiently transfected with FAM-tagged miR21 (Fig. 3d) and injected adjacent to the tumour nodules. Tumours were harvested 24 h after fibroblast injection and frozen sections were prepared. Confocal microscopy showed blue 4,6-diamidino-2-phenylindole signals of the round nuclei, indicating the presence of intact cells (Fig. 3e). Ovarian cancer cells were identified by red mCherry labelling, which appeared as granular structures in non-fixed frozen sections (Fig. 3f). Green FAM labelling signals, which represented individual miR21 molecules, were observed in the peripheral stromal cells as well as in some of the cancer cells (Fig. 3g,h). These results suggest that miR21 can be transferred from stromal fibroblasts to cancer cells *in vivo*.

As transforming growth factor (TGF-β), a well-known regulator of the cancer–stroma interaction, was reported to regulate miR21 expression in pulmonary artery smooth muscle cells^20^ and pulmonary fibroblasts^21^, we determined whether increased miR21 expression in ovarian cancer cells was due to TGF-β produced by the stromal cells, which upregulated endogenous miR21 in ovarian cancer cells rather than exosomal transfer. We treated ovarian cancer SKOV3 and OVCA432 cells and CAFs with TGF-β and quantified the miR21 expression levels in these cells by qRT–PCR analysis. The results showed that there were no significant increases in miR21 expression levels in the ovarian cancer cells (Supplementary Fig. 2b) or CAFs (Supplementary Fig. 2c). Thus, the increase in the miR21 expression level in cancer cells was primarily due to the direct transfer of miR21 from neighbouring stromal cells in the microenvironment via exosomes instead of TGF-β stimulation.

### miR21 shows no effect on cancer cell proliferation

To determine whether miR21 stimulates ovarian cancer cell proliferation *in vitro*, ovarian cancer SKOV3 and OVCA432 cells were incubated for 72 h after transient transfection of miR21 precursor or control miR. The relative cell number of SKOV3 and OVCA432 cells shows no significant changes with or without miR21 expression (Supplementary Fig. 3a).

To further evaluate the effect of miR21 on ovarian cancer cell proliferation *in vivo*, luciferase-labelled SKOV3ip ovarian cancer cells and ^miR21+/miR21+^MEF (miR21-MEF) or ^miR21−/miR21−^MEF (MEF) cells were subcutaneously injected into female BALB/c athymic nude mice, to establish tumours. The tumour volumes were measured and quantified 5 days post injection. The luciferase activity of the tumour established shows no significant changes between the miR21-MEF group and the MEF group (Supplementary Fig. 3b).

### miR21 stimulates cancer cell motility and invasion

Besides cell proliferation, we determined whether miR21 modulated other malignant phenotypes such as the motility and invasive potential of ovarian cancer cells. The ovarian cancer cells ALST, OVCA433 and SKOV3 were transfected with miR21 precursor (pre-miR21) or negative control (control miR) and seeded onto a transwell plate coated with type I collagen matrix. The cells were allowed to invade for 24 h. Invading cells were then fluorescently labelled with calcein and quantified. The results showed a significant increase in invasive potential in all three cell lines when they were transfected with miR21 compared with the mock transfectants (Supplementary Fig. 4a). An *in vitro* scratch assay was also performed to evaluate the effect of miR21 on cell motility. The results showed that all three cell lines demonstrated significantly faster wound closure rates in miR21-transfected cells than in mock-transfected cells, suggesting that miR21 enhanced the motility potential of ovarian cancer cells (Supplementary Fig. 4b).

### miR21 lowers chemosensitivity and apoptosis in cancer cells

**Figure 4:**
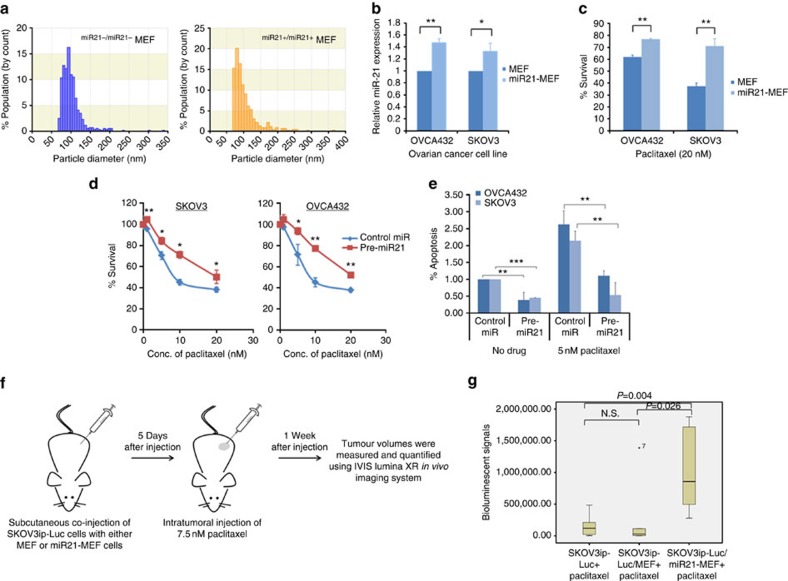
MiR21 enhances ovarian cancer cell chemoresistance and decreases apoptosis in ovarian cancer cells. (**a**) qNano analyses of exosomes isolated from ^miR21−/miR21−^MEFs and ^miR21+/miR21+^MEFs. A majority of the purified exosomes were <100 nm in diameter. (**b**) qRT–PCR analysis showing a significant increase in miR21 in SKOV3 and OVCA432 cells treated with ^miR21+/miR21+^MEF exosomes compared with those treated with ^miR21−/miR21−^MEF exosomes. The results were the average from at least three independent experiments. Mean±s.d.; **P<0.01 and *P<0.05; two-tailed Student's *t*-test. (**c**) Bar charts showing a significant decrease in cell number in SKOV3 and OVCA432 cells treated with ^miR21−/miR21−^MEF exosomes compared with those treated with ^miR21+/miR21+^MEF exosomes in the presence of 20 nM paclitaxel. The results were the average from at least three independent experiments. Mean±s.d.; **P<0.01; two-tailed Student's *t*-test. Effect of miR21 overexpression on (**d**) paclitaxel sensitivity and (**e**) paclitaxel-induced apoptosis in OVCA432 and SKOV3 ovarian cancer cells. After being transfected with miR21 precursor (pre-miR21) or negative control (control miR), the cells were incubated with paclitaxel for 3 days. Cell survival and apoptosis were measured by MTT assay and Cell Death Detection ELISA, respectively. The results were the average from at least three independent experiments. Mean±s.d.; ***P<0.001, **P<0.01 and *P<0.05; two-tailed Student's *t*-test. (**f**) A schematic diagram showing the use of the *in vivo* model to evaluate the effects of CAFs on the paclitaxel sensitivity of cancer cells. (**g**) Luciferase-labelled SKOV3ip ovarian cancer cells and ^miR21+/miR21+^MEF (miR21-MEF) or ^miR21−/miR21−^MEF (MEF) cells, or SKOV3ip cells alone were subcutaneously injected into female BALB/c athymic nude mice, to establish tumours. The tumour volumes were measured and quantified using the IVIS-Lumina XR *in vivo* imaging system after a 7-day intratumoral paclitaxel treatment. A box plot showing a significant decrease in luciferase activity in the miR21-MEF group (*n*=6) compared with the MEF group (*n*=6) after paclitaxel treatment (*P*=0.026; Mann–Whitney *U*-test) or the cancer cells alone group (*n*=6) after paclitaxel treatment (*P*=0.004; Mann–Whitney *U*-test). NS, not significant (*P*>0.05; Mann–Whitney *U*-test).

To determine whether exosomal miR21 confers chemoresistance in ovarian cancer cells, we isolated exosomes from ^miR21−/miR21−^MEFs and ^miR21+/miR21+^MEFs. They were quantified by qNano (Fig. 4a) and equal numbers of exosomes were added to two ovarian cancer cell lines, OVCA432 and SKOV3. A significantly higher level of miR21 was confirmed in ^miR21+/miR21+^MEF exosome-treated cells than in ^miR21−/miR21−^MEF exosome-treated cells (Fig. 4b). Both OVCA432 and SKOV3 cells were treated with 20 nM paclitaxel. The results showed a significant decrease in cell number in cells treated with ^miR21−/miR21−^MEF exosomes compared with those treated with ^miR21+/miR21+^MEFs exosomes (Fig. 4c).

To further evaluate the role of miR21 in chemoresistance, we directly transfected OVCA432 and SKOV3 ovarian cancer cells, which have low levels of endogenous miR21 expression, with miR21 precursor (pre-miR21) or the negative control and treated them with paclitaxel. The resulting miR21 expression levels in the transfected cells were comparable to the physiological level (Supplementary Fig. 5). The cells transfected with the miR21 precursor showed significantly lower chemosensitivity than did the mock transfectants (Fig. 4d). In addition, OVCA432 and SKOV3 cells transfected with the miR21 precursor had a lower rate of apoptosis than did the mock transfectants (Fig. 4e). Next, we determined whether overexpression of the other two most abundant miR21 variants that we identified had the same biological effect on ovarian cancer cells. Both miR21 mature 5′ super variant and precursor variant, together with the mature miR21, were transfected into SKOV3 and OVCA432 cells and treated with paclitaxel. All three miRNAs demonstrated a significant increase in IC_50_ in the transfected cells (Supplementary Fig. 6a), suggesting that the two miR21 variants have similar biological activities to those of the mature miR21.

To demonstrate the role of miR21 in chemoresistance in ovarian cancer cells *in vivo*, luciferase-labelled ovarian cancer SKOV3ip cells were co-injected with ^miR21+/miR21+^MEFs (miR21-MEF) or ^miR21−/miR21−^MEFs (MEFs) into female nude mice, followed by a sub-optimal dose of paclitaxel (7.5 nM). SKOV3ip cells alone were also injected as a negative control. We found that the luciferase activity of both the SKOV3ip cell-alone group and the MEF group were significantly lower than that of the miR21-MEF group after the same paclitaxel treatment, suggesting that tumour cells in the miR21-MEF group are more resistant to paclitaxel than those in the SKOV3ip cell-alone group and the MEF group (Fig. 4f,g).

### APAF1 is a direct downstream target of miR21 in cancer cells

**Figure 5:**
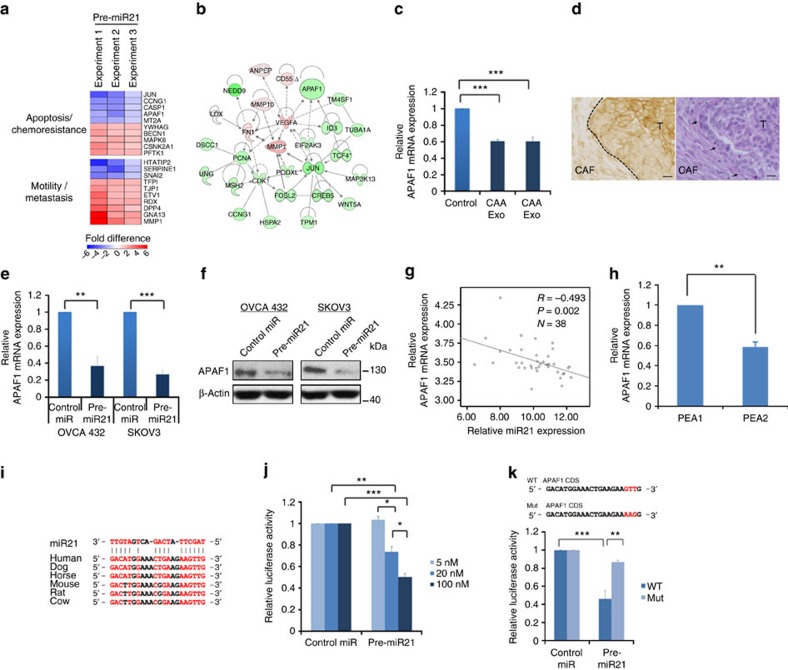
APAF1 is a miR21 direct target in ovarian cancer cells. (**a**) Transcriptome profiling of SKOV3 cells transfected with miR21 precursor or control miR. A heatmap shows the top 10 miR21 regulated genes related to chemoresistance and metastasis. (**b**) Pathway analysis showing chemoresistance-related genes that were upregulated (red) or downregulated (green) in miR21-transfected SKOV3 cells. (**c**) SKOV3 cells incubated with CAA- and CAF-derived exosomes showed lower APAF1 expression than did controls. The results were the average from at least three independent experiments. Mean±s.d.; ***P<0.001; two-tailed Student's *t*-test. (**d**) Immunolocalization of APAF1 on ovarian tumour tissue sections (*n*=5) demonstrated lower APAF1 level in the stromal–epithelial interface compared with that in the centre of the tumour tissues. A representative serial section of the tumour tissue was stained with haematoxylin and eosin (right panel). Dotted line and arrowheads indicate the stromal–epithelial interface between CAF and tumour (T). Scale bar, 10 μm. (**e**,**f**) Ovarian cancer cells transfected with miR21 precursor had lower APAF1 mRNA and protein levels than did controls. The results were the average from at least three independent experiments. Mean±s.d.; ***P<0.001 and **P<0.01; two-tailed Student's *t*-test. (**g**) The correlation between miR21 and APAF1 mRNA levels in microdissected ovarian cancer tissues was determined using a Spearman's correlation analysis. (**h**) Recurrent ovarian cancer PEA2 cells expressed lower APAF1 expression than did primary PEA1 cells. The results were the average from at least three independent experiments. Mean±s.d.; **P<0.01; two-tailed Student's *t*-test. (**i**) A consensus miR21-binding site was identified within the coding sequence of APAF1. (**j**) Co-transfection of pre-miR21 and luciferase vector with APAF1 coding sequence decreased the luciferase expression in SKOV3 cells in a dose-dependent manner. The results were the average from at least three independent experiments. Mean±s.d.; ***P<0.001, **P<0.01 and *P<0.05; two-tailed Student's *t*-test. (**k**) The luciferase activity was measured following co-transfection with either wild-type (WT) or mutated (Mut) APAF1 coding sequence vectors (mutated sequence shown in red in upper panel). The results were the average from at least three independent experiments. Mean±s.d.; ***P<0.001 and **P<0.01; two-tailed Student's *t*-test.

To delineate the molecular mechanisms underlying the role of miR21 in conferring chemoresistance in ovarian cancer cells, we performed transcriptome profiling on SKOV3 cells transfected with pre-miR21 or the pre-miR-negative control (GEO #GES51457). Pathway analyses identified a set of chemoresistance-associated genes (Fig. 5a,b and Supplementary Table 3). Among these genes, *APAF1*, which has been shown to be associated with chemoresistance and apoptosis^22^,^23^,^24^, was selected for further validation studies. To confirm that APAF1 can be downregulated by miR21 delivered in CAA- and CAF-derived exosomes, we incubated SKOV3 cells with exosomes isolated from CAAs and CAFs. qRT–PCR analyses showed that miR21 expression levels in SKOV3 cells incubated with CAA- and CAF-derived exosomes were significantly higher than in cells incubated with complete media only (Supplementary Fig. 2a). In contrast, APAF1 mRNA levels were significantly lower in SKOV3 cells incubated with exosomes derived from miR21-transfected CAAs and CAFs (*P*<0.001) (Fig. 5c). These data suggest that exosomes derived from miR21-transfected CAAs and CAFs can significantly increase miR21 and lower APAF1 expression in recipient ovarian cancer cells. This finding is further supported by the immunohistochemistry data that showed lower APAF1 expression in the stromal–epithelial interface (Fig. 5d), in some of the cases (*n*=5) which expressed high levels of stromal miR21 (Fig. 2e–g).

To determine whether APAF1 is a direct downstream target of miR21, we performed a qRT–PCR analysis of ovarian cancer OVCA432 and SKOV3 cells transfected with the miR21 precursor or the negative control, to evaluate its effect on APAF1 expression. The results showed significantly lower APAF1 expression in both cell lines transfected with the miR21 precursor than in the control (Fig. 5e). A similar effect was also observed in cells transfected with the two miR21 variants (miR21 mature 5′ super variant and precursor variant) (Supplementary Fig. 6b). A western blot analysis was then performed to show downregulation of the APAF1 protein in both cell lines (Fig. 5f). The inverse correlation between miR21 and APAF1 mRNA expression was further confirmed by qRT–PCR analyses of the epithelial components of 38 microdissected ovarian cancer tissue samples (*R*=−0.493, *P*=0.002) (Fig. 5g). In addition, PEA2 recurrent ovarian cancer cells, which showed significantly higher miR21 expression than did PEA1 primary cells (Fig. 2g), had significantly lower APAF1 mRNA expression than did PEA1 cells (Fig. 5h).

To determine whether APAF1 is a direct target of miR21, we determined the alignment between miR21 sequences and the full length of APAF1 using RNA22 software. The results showed that an APAF1 coding sequence (5′-GACATGGAAACTGAAGAAGTTG-3′) that had a significant homology among different species was a potential target for miR21 (Fig. 5i). Subsequently, the potential miR21 binding sequence was cloned into the luciferase vector and co-transfected into OVCA432 cells in the presence of different concentrations of pre-miR21. The results showed a significant dose-dependent decrease in luciferase activity in cells transfected with pre-miR21 (Fig. 5j). In addition, the effect of pre-miR21 on luciferase activity was abrogated when cells were transfected with a mutated APAF1 coding sequence (Fig. 5k). These data suggest that miR21 directly controls APAF1 mRNA expression through binding to the APAF1 coding sequence, and that APAF1 is a direct target of miR21.

### APAF1 mediates miR21-induced chemoresistance in cancer cells

**Figure 6:**
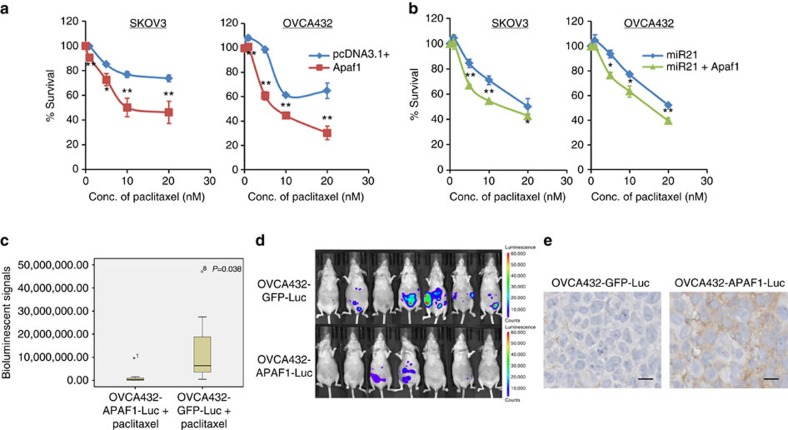
APAF1 mediates miR21-induced paclitaxel resistance. (**a**) Overexpression of APAF1 by full-length transfection increased paclitaxel sensitivity in ovarian cancer SKOV3 and OVCA432 cells. The results were the average from at least three independent experiments. Mean±s.d.; **P<0.01 and *P<0.05; two-tailed Student's *t*-test. (**b**) Co-transfection of miR21 precursor and full-length APAF1 decreased paclitaxel resistance compared with the co-transfection of pre-miR21 and the control vector in both SKOV3 and OVCA432 cells. The results were the average from at least three independent experiments. Mean±s.d.; **P<0.01 and *P<0.05; two-tailed Student's *t*-test. (**c**–**e**) APAF1 overexpression enhanced the paclitaxel sensitivity of ovarian cancer cells *in vivo*. APAF1 stably overexpressing ovarian cancer OVCA432 cells were generated using the lentiviral transduction method and were intraperitoneally injected into female BALB/c athymic nude mice, followed by paclitaxel treatment. The tumour volumes were measured and quantified using the IVIS-Lumina XR *in vivo* imaging system after a 2-week 5 mg kg^−1^ paclitaxel treatment. (**c**) Box plot showing a significant decrease in luciferase activity in the APAF1 overexpression group (*n*=7) compared with the control group (*n*=7) after paclitaxel treatment (*P*=0.038; Mann–Whitney *U*-test). (**d**) Representative images show a decrease in luminescence in the APAF1 overexpression group compared with the control group after paclitaxel treatment. (**e**) Immunolocalization of APAF1 on paraffinized sections of tumour tissues collected from mice demonstrated a higher APAF1 level in the APAF1 overexpression group (*n*=7) compared with the control group (*n*=7). Representative microscopic images were illustrated. Scale bar, 10 μm.

To determine whether high levels of APAF1 confer paclitaxel sensitivity to ovarian cancer cells, we transiently transfected SKOV3 and OVCA432 ovarian cancer cells with the full-length APAF1 construct or the control vector pcDNA3.1 and treated them with various concentrations of paclitaxel. Both cell lines demonstrated a significant decrease in IC_50_ in cells transfected with the APAF1 construct compared with the mock transfectants (Fig. 6a). These data suggest that APAF1 sensitized the ovarian cancer cells to paclitaxel treatment. To determine whether upregulation of APAF1 can abrogate miR21-conferred paclitaxel resistance in ovarian cancer cells, we transfected both SKOV3 and OVCA432 cells with pre-miR21 and APAF1 or with pre-miR21 and the empty vector. APAF1 partially abrogated paclitaxel resistance (Fig. 6b), suggesting that besides downregulation of AFAP1, other mechanisms are involved in mediating the effect of miR21 on paclitaxel resistance in ovarian cancer cells. The results were further confirmed in OVCA432 cells that were stably transfected with a doxycycline-inducible APAF1 construct (Supplementary Fig. 7).

To evaluate the effect of APAF1 on paclitaxel resistance in ovarian cancer cells *in vivo*, we established OVCA432 cells stably expressing doxycycline-inducible APAF1 or the control vector and injected them intraperitoneally into female nude mice, followed by paclitaxel treatment. The results showed that the luciferase activity of the APAF1-overexpressing group was significantly lower than that of the control group after the same paclitaxel treatment (Fig. 6c,d). Elevation of APAF1 expression in the tumour tissues from the APAF1-overexpressing group was confirmed using immunohistochemical analysis (Fig. 6e). These data suggest that APAF1 confers paclitaxel resistance in ovarian cancer cells.

## Discussion

In this study, we showed, for the first time, that CAAs and CAFs express significantly higher levels of miR21 isomiRs than do ovarian cancer cells, and that the exosomal transfer of miR21 isomiRs from CAAs or CAFs to neighbouring cancer cells can increase the chemoresistance of these cells to paclitaxel through the downregulation of miR21's direct target APAF1 (Supplementary Fig. 8).

The bidirectional signalling mediated by secreted proteins that occurs between ovarian cancer cells and the omental fibroblasts and adipocytes not only effectively ‘reprogrammes' fibroblasts and adipocytes to create an optimal microenvironment for tumour implantation but also confers a more aggressive phenotype to the cancer cells that allows them to adapt to their new microenvironment^16^,^25^. Recent studies have shown that cells can communicate through the exchange of bioactive molecules via microvesicles. These small vesicles include ectosomes (100–1,000 nm) and exosomes (30–100 nm), which are shed or secreted by almost all cell types^4^. Exosomes are endosome-derived microvesicles that are secreted as part of multivesicular bodies into the extracellular space. Secreted exosomes can be engulfed by tissues locally or carried in biological fluids to distant sites in the body^3^. Intercellular communication can be initiated through interactions between circulating exosomes and the transmembrane proteins of target cells. Target cells engulf exosomes through endocytosis, resulting in the intracellular release of bioactive molecules, which include membrane proteins, cytoplasmic proteins and lipids.

Recent studies have shown that exosomes also contain non-coding RNAs such as miRNAs^14^. MiR21 has been identified in exosomes derived from breast cancer cells^26^, dendritic cells^27^, T cells^27^ and serum samples^12^. Using Ion Torrent next-generation sequencing of miRNA in exosomes, we identified significantly larger copy numbers of miR21 isomiRs, in particular the mature 5′ and 5′ super variants, in exosomes isolated from CAFs and CAAs than in those isolated from ovarian cancer cells. This finding was probably due to higher endogenous miR21 expression in CAAs and CAFs than in cancer cells, as we confirmed by qRT–PCR analyses of microdissected tissue samples and *in situ* localization of miR21 in tissue sections. Significantly lower miR21 expression levels were detected in both normal fibroblasts and adipocytes than in ovarian cancer cells by a qRT–PCR analysis of microdissected tissue samples and *in situ* hybridization. However, miR21 expression levels in ovarian cancer cells and normal stromal cells *in vitro* were found to be comparable. The discrepancy may be due to miR21 being upregulated when quiescent normal stromal cells, in particular fibroblasts isolated from tissue samples, grow in culture. In addition, the lack of miR21 transfer from neighbouring CAFs or CAAs may lead to lower miR21 expression in cultured ovarian cancer cells.

By incubating ovarian cancer cells with purified exosomes from CAFs transfected with labelled miR21, we confirmed that miR21 was successfully transferred to the cancer cells. The transfer of miR21 from CAFs to cancer cells was further confirmed by co-culturing labelled miR21-transfected CAFs with ovarian cancer cells both *in vitro* and *in vivo*. Increased miR21 expression in cancer cells in the stroma–cancer interface also suggests that miR21 can be transferred from the stromal cells to the neighbouring cancer cells.

miRNAs are a family of short (20–24 nt) non-coding RNAs that posttranscriptionally regulate gene expression in multicellular organisms by affecting both the stability and translation of mRNAs^28^. MiR21 expression has been linked to resistance to a variety of chemotherapeutic agents in both solid and haematologic tumours. By silencing the expression of tumour suppressor genes including *PTEN*, *PDCD4* and *p21*, miR21 overexpression promotes cell survival and confers resistance to tamoxifen, faslodex and topotecan in breast cancer^29^. Furthermore, miR21 expression is associated with resistance to gemcitabine, 5-fluorouracil and docetaxel in pancreatic, colorectal and prostate cancers, respectively^30^,^31^,^32^. miR21 can be transferred by exosomes from stromal cells to cancer cells and can decrease paclitaxel sensitivity, as shown in this study, suggesting that increased miR21 expression in ovarian cancer cells, in particular those in the stroma–cancer interface, may confer a more aggressive and chemoresistant phenotype in those cells than in those located inside the tumour mass.

A transcriptome profiling analysis and subsequent validation studies demonstrated a significant decrease in APAF1 in miR21-transfected ovarian cancer cells. APAF1 binds with cytochrome *c* and dATP to form an apoptosome, which in turn activates caspases 9 and 3 to initiate apoptosis^33^,^34^. APAF1 deficiency has been documented to confer chemoresistance in several cancer types^35^. Thus, downregulation of APAF1 by miR21 may increase paclitaxel resistance in ovarian cancer cells. This was confirmed by co-transfecting ovarian cancer cells with both miR21 and APAF1 in the presence of paclitaxel. However, APAF1 cannot completely rescue the effect of miR21 on paclitaxel resistance, suggesting that other miR21 target genes are involved. APAF1 is not a known direct target of miR21; we also failed to demonstrate any sequences in miR21 that were complementary to the 3′-untranslated region of the APAF1 mRNA. Instead, we showed, for the first time, that an APAF1 coding sequence was a potential target for miR21. Further studies confirmed that miR21 directly controls APAF1 mRNA expression through binding to the APAF1 coding sequence, and that APAF1 is a direct target of miR21.

Our transcriptome analysis of miR21-transfected ovarian cancer cells showed upregulation of MMP1, which may lead to an increase in the invasion potential of ovarian cancer cells, as we observed in our *in vitro* studies. MMP1 is a member of the MMP family, which is associated with invasion and metastasis^36^. MMP1 is not a known target of miR21; however, a pathway analysis showed that multiple transcription factors that have been shown to regulate MMP1 were up- or downregulated in miR21-transfected SKOV3 cells. Among them, smad7 has been shown to be a direct target of miR21 (ref. 37) and a negative regulator of TGF-induced MMP1 expression^38^, suggesting that miR21 regulates MMP1 expression indirectly by targeting genes in the TGF signalling pathways.

In conclusion, our results show that exosomal miR21 can confer chemoresistance and an aggressive phenotype in ovarian cancer cells through its transfer from neighbouring stromal cells, suggesting that preventing the exosomal transfer of miR21 from stromal cells is a new strategy for suppressing ovarian cancer growth. In addition, the identification of APAF1 as a direct target of miR21 and APAF1 as a mediator of miR21 for conferring chemoresistance in ovarian cancer suggests that strategies based on the upregulation of APAF1 in ovarian cancer cells can be used to sensitize ovarian cancer cells to paclitaxel treatment.

## Methods

### Microdissection of tissue samples

Ovarian tissue samples were obtained from the ovarian cancer repository of the Department of Gynecologic Oncology and Reproductive Medicine under protocols approved by the institutional review board of The University of Texas MD Anderson Cancer Center (Houston, Texas). Normal omental tissue samples were obtained from patients with benign gynaecologic diseases in the Department of Obstetrics and Gynecology under protocols approved by the institutional review board of Baylor College of Medicine. Informed consent was obtained from all patients. Stromal and epithelial components of normal and malignant ovarian tissues were obtained by microdissection. Microdissection was conducted by fixing tissue sections in 70% ethanol and staining them with 1% methyl greenm to visualize the histologic features. During dissection, the areas of interest in the sections were carefully outlined. Areas with immune cell and blood vessel infiltration were excluded, to minimize contamination.

### Isolation of primary fibroblasts and adipocytes

Normal and CAF primary cultures were derived from normal ovaries from patients with benign gynaecologic diseases and from high-grade serous ovarian cancer tissue, respectively, using the explant technique. In brief, tissue samples were cut into 1-mm^3^ fragments and seeded into T25 tissue culture flasks with 2-ml tissue culture media. After 1 week, an additional 3 ml of culture media were added. Once the flask became 50% confluent, the explants were removed and the epithelial cells were scraped from the flasks under the microscope using a cell scraper. Cells were then subcultured into two T25 flasks. After two to three passages, the percentage of fibroblasts was determined by immunohistochemistry.

Normal adipocytes and CAA primary cultures were derived from the omental tissues of patients with benign gynaecologic diseases and high-grade serous ovarian cancer, respectively. Adipose tissue was first digested with collagenase (Sigma-Aldrich Co., St Louis, MO, USA) in HEPES. The digested tissues were then centrifuged. The pellet containing preadipocytes, fibroblasts and red blood cells was removed, while the top fatty layer was washed in fresh DMEM/F12 medium twice, with centrifugation, before being transferred to the cell culture flasks. The flasks were fully filled with 10% fetal bovine serum (FBS) DMEM/F12 medium and were inverted so that the bottom of the flask was on top. The floating mature adipocytes were attached to the upper portion of the flasks, while any fibroblast-like cells were sunk to the bottom. After 5–7 days to allow sufficient attachment of the mature adipocytes, the media inside were removed and replaced with 5 ml of fresh media. The flasks were then re-inverted for normal observation and manipulation^39^. The mature adipocytes isolated were characterized using Oil Red O staining.

### Cell lines and culture conditions

The human ovarian cancer cell lines A2780 (Sigma-Aldrich Co.), ALST (gifts from Dr Michael Birrer's laboratory at the Massachusetts General Hospital), OVCA432, OVCA433 (gifts from Dr Robert Bast's laboratory at the MD Anderson Cancer Center), OVCAR5 (gifts from Dr Gordon Mills's laboratory at the MD Anderson Cancer Center), HeyA8, HeyA8-MDR, SKOV3ip, SKOV3-TR (gifts from Dr Anil Sood's laboratory at the MD Anderson Cancer Center) and SKOV3 (American Type Culture Collection) were maintained in RPMI 1640 medium supplemented with 10% FBS, 2 mM glutamine and penicillin/streptomycin (Life Technologies Corp., Grand Island, NY, USA). The human ovarian cancer cell lines PEA1 and PEA2 (European Collection of Authenticated Cell Cultures) were maintained in RPMI 1640 medium supplemented with 10% FBS, 2 nM glutamine, penicillin/streptomycin and 1% sodium pyruvate (Sigma-Aldrich Co.). Normal adipocytes and CAAs were established from the omental tissue removed from patients with benign gynaecologic diseases and ovarian cancer, respectively. They were cultured in DMEM medium supplemented with 10% FBS. Normal ovarian fibroblasts and CAFs were established from normal ovaries and ovarian cancer tissue removed from patients with benign gynaecologic diseases and ovarian cancer, respectively. They were cultured in 1:1 MCDB105/199 medium (Sigma-Aldrich Co.) supplemented with 10% FBS, 1 ng ml^−1^ epidermal growth factor and penicillin/streptomycin (Life Technologies Corp.). MEFs (gifts from Dr Yong Li's laboratory at the University of Louisville)^40^ were cultured in RPMI 1640, supplemented with 10% FBS and non-essential amino acid (Life Technologies Corp.). pEZX-miR21 plasmids (GeneCopoeia, Rockville, MD, USA) were stably transfected into ^miR21−/miR21−^MEF cells to overexpress miR21. The miR21 stably overexpressed ^miR21−/miR21−^MEF cells were annotated as ^miR21+/miR21+^MEF cells. Empty pEZX vectors were also stably transfected into ^miR21−/miR21−^MEF cells as negative controls. All cells were tested negative for mycoplasma contamination and were authenticated by short tandem repeat profiling in the Characterized Cell Line Core at the University of Texas MD Anderson Cancer Center.

### Isolation of exosomes

After cell cultures reached 90% confluency, cells were washed with PBS and incubated with freshly prepared complete medium containing exosome-free FBS for 48 h. Exosomes were isolated from the conditioned medium by differential centrifugation. In brief, conditioned medium was centrifuged at 300 *g* for 10 min and then at 2,000 *g* for 20 min at 4 °C to remove cells, followed by filtration through a 0.22-μm filter to remove cell debris. Exosomes were pelleted by ultracentrifugation at 100,000 *g* for 90 min. They were resuspended in PBS and collected by ultracentrifugation again at 100,000 *g* for 90 min. The size and concentration of the exosomes were quantified by qNano (Izon Science, New Zealand) and exosomes were ready for RNA/protein extraction or cell treatment.

### Transmission electron microscopy

For electron microscopy analysis, exosomes were prepared by mixing them with an equal volume of 4% paraformaldehyde. Samples were deposited onto Formvar-carbon-coated electron microscopy grids, washed with PBS and further fixed with 1% glutaraldehyde for 5 min. Samples were contrasted first in a solution of uranyl oxalate, pH 7, for 5 min and then contrasted and embedded in a mixture of 4% uranyl acetate and 2% methyl cellulose for 10 min on ice. The samples were observed using a JEM 1010 transmission electron microscope (JEOL USA, Inc., Peabody, MA) at 80 kV.

### Western blot analysis

Cell extracts were prepared in RIPA buffer (20 mM sodium phosphate, 150 mM NaCl pH 7.4, 1% Nonidet P-40, 0.1% SDS and 0.5% deoxycholic acid) containing complete protease inhibitor mixture (Roche). Proteins were separated on SDS–polyacrylamide gels and electrophoretically transferred to an Immobilon polyvinylidene fluoride membrane (EMD Millipore, Billerica, MA, USA). The membranes were incubated with primary antibodies overnight at 4 °C, followed by incubation with appropriate horseradish peroxidase-conjugated secondary antibodies at 1:10,000 dilution (Thermo Fisher Scientific, Grand Island, NY, USA) for 1 h at ambient temperature. Signals were developed using ECL chemiluminescence detection reagents (GE Healthcare Life Sciences, Pittsburgh, PA, USA) and visualized on X-ray film (Fujifilm, Tokyo, Japan). The following primary antibodies were used for western blotting: anti-CD63 (1:1,000; clone RFAC4, EMD Millipore), anti-GM130 (1:1,000; 610822, BD Biosciences, San Jose, CA, USA), anti-HSP70 (1:1,000; MAB3516, EMD Millipore), anti-APAF1 (1:1,000; ab2, Sigma-Aldrich Co.) and anti-β-actin (1:5,000; clone AC-15, Sigma-Aldrich Co.). Original images of all blots are supplied as Supplementary Figs 9 and 10.

### RNA sequencing using Ion Torrent

Exosomes were isolated from three CAF and two CAA primary cultures and four ovarian cancer cell lines (A2780, HeyA8, OVCA433 and SKOV3) following the procedure as described above. Total RNAs were extracted from purified exosomes using the RNAqueous-Micro kit (Thermo Fisher Scientific) and the amount and quality of small RNA in the total RNA samples were determined using the Agilent small RNA kit on an Agilent 2100 Bioanalyzer. Five nanograms of small RNAs were subjected to library construction using the Ion Total RNA-Seq kit v2 (Life Technologies Corp.) according to the small RNA protocol. In brief, the RNA was first hybridized and ligated with ion adaptors at 16 °C for 16 h, followed by a reverse-transcription reaction. Size selection of the complementary DNAs was performed with a Novex (Life Technologies Corp.) 10% TBE-urea pre-cast gel. After being stained with SYBR Gold nucleic acid, the DNA fragments of 60–80 nt were cut from the gel and subjected to PCR amplification for 18 cycles. The amplified product was purified using the PureLink PCR micro kit (Life Technologies Corp.) and the yield and size distribution were analysed on an Agilent 2100 Bioanalyzer with the Agilent DNA 1000 kit. Emulsion PCR and ion sphere particle (ISP) enrichment was performed using the Ion Xpress Template kit (Life Technologies Corp.) according to the manufacturer's instructions. In brief, 140 × 10^6^ template molecules were added to the emulsion PCR master mix and the emulsion was generated using the IKA Ultra-Turrax Tube Drive (Life Technologies Corp.). The mixed emulsion was transferred to a 96-well PCR plate and amplified using a thermal cycler. ISPs were recovered and template-positive ISPs were enriched using Dynabeads MyOne Streptavidin C1 beads (Life Technologies Corp.). Quality control of ISPs was performed using a Qubit 2.0 Fluorometer (Life Technologies Corp.) and the sample was prepared for sequencing using the Ion sequencing kit protocol (Life Technologies Corp.). The complete sample was loaded on an Ion 314 chip and sequenced on the Ion Torrent Personal Genomic Machine for 65 cycles.

Ion Torrent reads were collected using Ion Torrent Suite software, which also sorted the data according to the barcodes. The software scores the quality of the reads by assigning Q17 and Q20 scores according to the quality scoring computation. Fastq files from the Ion Torrent server were imported to the CLC Genomics Workbench (version 5.1; Life Technologies Corp.). Sequence reads were trimmed to remove reads with ambiguous nucleotides and to create a sequence length of 15–55 nucleotides. Trimmed sequence reads were aligned to an miRNA database (miRBase, release 18). Mapped reads were annotated and counted. The median number of reads was 182,000 for each sample. Ion Torrent reads were then normalized to the number of cells in each cell line used for exosome collection and presented as the total reads per 1,000 cells.

### Quantitative RT–PCR analysis

Total RNA was extracted from cultured cells using TRI reagent (Molecular Research Center, Cincinnati, OH, USA) or from tissue samples and purified exosomes using the RNAqueous-Micro kit (Thermo Fisher Scientific).

For exosomal miR21 expression analysis, 0.5 μg of total RNA was first reverse transcribed using the QuantiMir RT kit (System Biosciences, Camarillo, CA, USA). qRT–PCR analysis was performed using iTaq Universal SYBR green super mix (Bio-Rad Laboratories Inc., Hercules, CA, USA) on a CFX Touch real-time PCR detection system (Bio-Rad Laboratories Inc.). The sequence of mature 5′ conventional miR21 (5′-TAGCTTATCAGACTGATGTTGA-3′), mature 5′-miR21 super variant (5′-TAGCTTATCAGACTGATGTTGACA-3′) or mature 5′-miR21 precursor variant (5′-TAGCTTATCAGACTGATGTTGACTA-3′) were used as the forward primer and the 3′ universal reverse primer was provided by the QuantiMir RT kit.

For cellular miR21 expression analysis, 5 ng of total RNA was first reverse transcribed using the TaqMan microRNA reverse transcription kit (Thermo Fisher Scientific) and miR21 (000397) and U6 (001973) snRNA-specific primers and probes, and then quantified using real-time PCR on a CFX96 Touch real-time PCR detection system (Bio-Rad Laboratories Inc.). The relative standard curve method (2^−ΔΔCt^) was used to determine relative miR21 expression using U6 snRNA as the reference.

For the mRNA expression analysis, 0.5 μg of total RNA was used to synthesize the first strand of cDNA using the ImProm-II Reverse Transcription System (Promega Corp., Madison, WI, USA). The expression of APAF1 was determined by multiplexing quantitative PCR (TaqMan Gene Expression Assay) using FAM-labelled APAF1 (Hs00559441_m1) together with VIC-labelled glyceraldehyde 3-phosphate dehydrogenase-specific TaqMan probes and primers. The relative standard curve method (2^−ΔΔCt^) was used to determine the relative mRNA expression, using glyceraldehyde 3-phosphate dehydrogenase as the reference.

### *In situ* hybridization of miR21

*In situ* hybridization was performed to determine the expression of miR21 using miRCURY LNA microRNA Detection, Optimization Kit 2 (miR-21) (Exiqon, Denmark) according to the manufacturer's protocol. In brief, 4-μm-thick sections of formalin-fixed, paraffin-embedded tissues were deparaffinized and digested with 15 μg ml^−1^ proteinase-K at 37 °C for 10 min. Slides were hybridized with 40 nM miR21 or scramble probes in an incubation chamber at 53 °C for 1 h, followed by stringent washes with SSC buffers at 53 °C. For immunodetection, slides were blocked with digoxigenin blocking reagent (Roche, Switzerland) in maleic acid buffer containing 2% sheep serum at ambient temperature for 15 min and then incubated with sheep anti-digoxigenin conjugated to alkaline phosphatase (diluted 1:800 in blocking reagent (Roche)) at ambient temperature for 60 min. Nitro-blue tetrazolium and 5-bromo-4-chloro-3′-indolyphosphate reaction mixture (Roche) with 0.2 mM levamisole (Sigma-Aldrich Co.) were applied to the section for colour development at 30 °C. After 2 h of incubation, the reaction was stopped with KTBT buffer. The slides were then counterstained with FastRed nuclear staining reagent, rinsed with tap water, dehydrated and mounted for microscopy. Five random microscope fields per section were evaluated at × 20 original magnification and the integral optical density of every visual field was calculated. For each patient, the same procedure was carried out using a 0.1-nM U6 snRNA probe to confirm the RNA integrity.

### *In vitro* detection of miR21 transfer

SKOV3ip cells were stably transfected with red mCherry (in pRsetB vector). For the co-culture experiment, CAFs and CAAs were grown on the 0.4 μm pore size transwell (Thermo Fisher Scientific) and transfected with 10 nM green fluorescent 3′ FAM-labelled miR-21 oligo (Sigma-Aldrich Co.) for 24 h. Cells were then put together with red fluorescent mCherry-labelled ovarian cancer SKOV3ip cells that had been grown on the cover slips in the bottom well of the transwell. After 24 h, the SKOV3ip cells were fixed with 4% paraformaldehyde. Fluorescent microscopy was used to detect the green signals in the SKOV3ip cells.

For the exosome treatment experiment, CAFs and CAAs were transfected with 10 nM green fluorescent FAM-labelled miR21 for 24 h. Cells were washed with PBS and incubated with freshly prepared complete medium containing exosome-free FBS for 48 h. CAF- and CAA-conditioned medium was collected and exosomes were isolated from the conditioned medium by differential centrifugation following the procedure described above. The pellet was suspended in serum-free medium and used to treat red fluorescently labelled ovarian cancer SKOV3ip cells grown on cover slips. After 24 h, the SKOV3ip cells were fixed with 4% paraformaldehyde and the nuclei were stained with 4,6-diamidino-2-phenylindole blue. SP5 confocal microscopy was used to detect the green signals in the SKOV3ip cells.

### *In vivo* detection of miR21 transfer

Red mCherry-labelled ovarian cancer SKOV3ip cells (2 × 10^6^) were injected subcutaneously into female BALB/c athymic nude mice at the age of 6 weeks, to establish tumours. After 1 week, ^miR21−/miR21−^MEFs transfected with 10 nM green fluorescent 3′ FAM-labelled miR-21 oligo (Sigma-Aldrich Co.) were injected intratumorally. Mice were killed using carbon dioxide chamber followed by cervical dislocation after 24 h and tumours were harvested and prepared for frozen sectioning. The frozen sections were unfixed and mounted with antifade reagent. Confocal microscopy was used to detect the green signals in the SKOV3ip cells. The described animal procedures have been reviewed and approved by the institutional animal care and use committee of the MD Anderson Cancer Center.

### MTT assay

SKOV3 and OVCA432 cells were seeded in a 96-well plate and then subjected to transient transfection for 24 h. After 72 h of paclitaxel treatment, the cells were incubated with 50 μl of 1 mg ml^−1^ MTT (3-(4,5-dimethylthiazol-2-yl)-2,5-diphenyltetrazolium bromide) (Sigma-Aldrich Co.) in PBS for 3 h. The formazan that formed was then solubilized by adding 150 μl of dimethyl sulfoxide. Absorbance was read at 570 nm using a FLUOstar Galaxy plate reader (BMG Labtech, Offenburg, Germany).

### Apoptosis assay

Apoptosis was measured using the Cell Death Detection ELISA^PLUS^ kit (Roche Applied Science, Indianapolis, IN, USA) following the manufacturer's protocol. In brief, cells were incubated with the lysis buffer at ambient temperature for 30 min, followed by centrifugation at 200 *g* for 10 min. The supernatant (20 μl) was incubated with 80 μl of anti-histone-biotin and anti-DNA-peroxidase reagent in a streptavidin-coated 96-well microtitre plate for 2 h at ambient temperature, with gentle shaking. The amount of peroxidase retained in the immunocomplex was photometrically determined with 2,2′-azino-di-[3-ethylbenzthiazoline sulfonate (6)] diammonium salt as a substrate. Absorbance was measured at 405 nm using a FLUOstar Galaxy plate reader. Data were expressed as the relative percentage of DNA fragmentation.

### Effect of miR21 and APAF1 on paclitaxel sensitivity *in vitro*

SKOV3 and OVCA432 cells were treated with 20 nM paclitaxel for 72 h after incubation with equal number of exosomes from ^miR21−/miR21−^MEF or ^miR21+/miR21+^MEF for 24 h. Cell viability was measured using MTT assay as described above.

SKOV3 and OVCA432 cells were transiently transfected with miR21 precursor or control miR (Thermo Fisher Scientific) for 24 h. Transfected cells were then subjected to a 72-h treatment of paclitaxel from 0 to 20 or 5 nM for the subsequent MTT assay or apoptosis assay, respectively.

SKOV3 and OVCA432 cells were transiently transfected with full-length Apaf1 in pcDNA3.1+ vector or empty pcDNA3.1+ vector for 24 h. Paclitaxel (0–20 nM) were then added to the transfected cells for 72 h. Cell viability was measured using MTT assay as described above.

### Effect of miR21 and APAF1 on paclitaxel sensitivity *in vivo*

SKOV3ip cells were labelled with luciferase (in pLKO vector) (Sigma-Aldrich Co.) using a lentiviral transduction method. Luciferase-labelled SKOV3ip cells (2 × 10^6^) alone or SKOV3ip cells (2 × 10^6^) with ^miR21+/miR21+^MEF (miR21-MEF) or ^miR21−/miR21−^MEF (MEF) cells (2 × 10^6^) were subcutaneously co-injected into female BALB/c athymic nude mice at the age of 6 weeks for 5 days, to establish tumours. The tumour volumes were measured and quantified using the IVIS-Lumina XR *in vivo* imaging system (Caliper Life Sciences, Inc., Hopkinton, MA, USA) after 7 days of treatment with 7.5 nM intratumoral paclitaxel. A full-length APAF1 open reading frame was subcloned into the doxycycline-inducible pInducer vector. APAF1 stably overexpressing ovarian cancer OVCA432 cells were generated using a lentiviral transduction method and intraperitoneally injected into female BALB/c athymic nude mice at the age of 6 weeks once for 1 week, followed by 5 mg kg^−1^ paclitaxel once a week for 2 weeks. Doxycycline-HCl (Sigma-Aldrich Co.) was dissolved in the drinking water to a final concentration of 2 mg ml^−1^, with 5% sucrose. The tumour volumes were measured and quantified using the IVIS-Lumina XR *in vivo* imaging system (Caliper Life Sciences, Inc.).

The described animal procedures have been reviewed and approved by the institutional animal care and use committee of the University of Texas MD Anderson Cancer Center.

### Microarray analysis

SKOV3 cells were transfected with either 5 nM miR21 precursor or negative control precursor miRNA for 24 h, and total RNA was isolated using TRI reagent (Molecular Research Center). Purified RNA samples were amplified and labelled using the MessageAmp Premier RNA amplification kit (Thermo Fisher Scientific) and then hybridized onto Affymetrix GeneChip Human Genome U133 Plus 2.0 microarrays according to the manufacturer's protocol. The arrays were washed and stained using the Affymetrix Fluidics Station 450 and GeneChip hybridization, washing and staining kit (Affymetrix Inc., Santa Clara, CA, USA). Scanning of the arrays was performed in the Cancer Genomics Core Laboratory at MD Anderson using the GeneChip Scanner 3000 7G (Affymetrix, Inc.). A heatmap was generated using dChip software (Affymetrix, Inc.). Pathway analyses of the data sets were performed using Ingenuity Pathway Software (Ingenuity Systems, Redwood City, CA, USA.

### Immunohistochemical analysis

Immunolocalization of APAF1 was performed using the tumour sections obtained from ovarian cancer patients or female BALB/c athymic nude mice injected with ovarian cancer OVCA432 cells that had been stably transfected with the doxycycline-inducible *APAF1* gene (OVCA432-APAF1-Luc) or with the control (OVCA432-GFP-Luc). Slides containing the sections were stained with commercially available anti-APAF1 (1:500; HPA031373) antibodies (Sigma-Aldrich Co.). Target protein expression was visualized using a Betazoid 3,3′-diaminobenzidine chromogen kit (Biocare Medical, Concord, CA, USA).

### Construction of APAF1 reporter vectors

The DNA fragment containing the predicted miR21-binding site was amplified from SKOV3 genomic DNA using the following primers: 5′-ATTAAGCTTGAGGCTCTAGATGAAGCCATGT-3′ (forward) and 5′-TTCACTAGTCCTGAAGCTGGCTGCAATTC-3′ (reverse). PCR products were subsequently cloned into the HindIII and SpeI sites of the pMIR-REPORT miRNA expression reporter vector (Thermo Fisher Scientific) and the integrity of constructs was confirmed by DNA sequencing. Site-directed mutagenesis was carried out to introduce mutations into the putative miR21-binding site using the QuikChange II site-directed kit (Stratagene, La Jolla, CA, USA). The mutation-containing primers were 5′-CTGGGACATGGAAACTGAAGAAAAGGAAGACATACTGCAGGAGTTTG-3′ (sense) and 5′-CAAACTCCTGCAGTATGTCTTCCTTTTCTTCAGTTTCCATGTCCCAG-3′ (antisense).

### Luciferase reporter gene activity assay

SKOV3 cells were seeded onto 24-well plates 1 day before transfection. The cells were transfected with 0.4 μg of the APAF1 reporter construct containing the predicted miR21-binding sites, together with 5, 20 and 100 nM miR21 precursor using Lipofectamine 2000 (Thermo Fisher Scientific). pRL-TK (1:20), which encodes *Renilla* luciferase, was included in all transfections to normalize transfection efficiency. The cells were washed and lysed with the passive lysis buffer from the Dual-Luciferase Reporter Assay System (Promega Corp.) 24 h after transfection. Luciferase activity was measured in each cell lysate using a FLUOstar Galaxy plate reader. Relative luciferase activity was first normalized with *Renilla* luciferase activity and then compared with those of the respective control.

### Effect of TGF-β on miR21 expression

Ovarian cancer SKOV3 and OVCA432 cells and CAFs were treated with 5 ng ml^−1^ TGF-β1 (Thermo Fisher Scientific) for 24 h before RNA extraction. The relative miR21 expression in cells were determined using the qRT–PCR analysis following the procedure as described above.

### Matrigel invasion assay

Matrigel (3.2 mg ml^−1^; BD Biosciences) in serum-free medium was added to each Transwell polycarbonate membrane insert (8-μm pore size, 6.5-mm diameter; Corning, Corning, NY, USA) and allowed to dry for 1 h at 37 °C. The cells (5 × 10^4^) were suspended in 140 μl of serum-free medium and seeded onto the upper chambers of the Transwell. Medium with 20% FBS (270 μl) was placed in the lower chambers. After 24 h of incubation, non-invading cells in the Matrigel were removed by wiping with a cotton swab. Invading cells at the Transwell membrane were green fluorescently labelled with calcein (Thermo Fisher Scientific). Cells from five random fields were counted at × 20 original magnification.

### *In vitro* wound-healing assay

Cells were seeded in 24-well plates to near confluence. Wounds were created by scratching the cell monolayer with a 200-μl pipette tip and cultures were washed with serum-free medium to remove detached cells. The wound healing was carried out in complete medium and was photographed after 0 and 14 h. To quantify cell migration, the wound width from ten different positions was measured at each time point and the migration distance was the difference in width between 0 and 14 h. The migration distance of each sample was first normalized to the initial wound width and then compared with the control sample. The experiment was repeated three times with similar results.

### Statistical analysis

SPSS software version 17 (IBM Corp., Armonk, NY, USA) was used to perform statistical tests. Data are presented as the mean±s.d. A two-tailed Student's *t*-test was used to test the differences in sample means for data with normally distributed means. Mann–Whitney *U*-test was used for non-parametric data. The correlation between mRNA expression levels in samples was determined using a Spearman's correlation analysis. A *P*-value of <0.05 was considered to be statistically significant.

## Additional information

**Accession codes:** Data files from the Ion Torrent RNA-sequencing analysis and the transcriptome profiling analysis were deposited in the Gene Expression Omnibus Data Bank (GEO; National Cancer for Biotechnology Information, Bethesda, MD, USA), respectively, under the accession codes GSE77318 and GSE51457.

**How to cite this article:** Au Yeung, C. L. *et al*. Exosomal transfer of stroma-derived miR21 confers paclitaxel resistance in ovarian cancer cells through targeting APAF1. *Nat. Commun.* 7:11150 doi: 10.1038/ncomms11150 (2016).

## Supplementary Material

Supplementary Information Supplementary Figures 1-10 and Supplementary Tables 1-3

Supplementary Data 1 Data sheet showing the average number of sequencing reads for each cell type normalized by counts per 1000 cells

Supplementary Data 2 Data sheet showing the average number of sequencing reads of miR21 isomiRs for each cell type normalized by counts per 1000 cells

## Figures and Tables

**Table 1:** Mean number of isomiR and miR21 isomiR species and total number of isomiRs and miR21 isomiRs per 1,000 cells in various cell types.

	OVCA (*n*=4)	NF (*n*=2)	CAF (*n*=3)	NA (*n*=2)	CAA (*n*=2)
Number of isomiR species	1,434	688	989	700	1,174
Total number of isomiRs per 1,000 cells	20,214	57,173	30,192	4,459,991	48,233,956
Number of miR21 isomiR species	46	34	55	16	33
Total number of miR21 isomiRs per 1,000 cells	4,091	20,250	12,240	677,042	10,022,035

CAA, cancer-associated omental adipocytes; CAF, cancer-associated fibroblasts; NA, normal omental adipocytes; NF, normal ovarian fibroblasts; OVCA, ovarian cancer cells.
