# Supplementary material for: Exosomal transfer of stroma-derived miR21 confers paclitaxel resistance in ovarian cancer cells through targeting APAF1
Source: Nat Commun. 2016 Mar 29;7:11150. doi: 10.1038/ncomms11150 (PMC4820618; doi:10.1038/ncomms11150)
Supplement: Supplementary Information — Supplementary Figures 1-10 and Supplementary Tables 1-3 [file ncomms11150-s1.pdf]

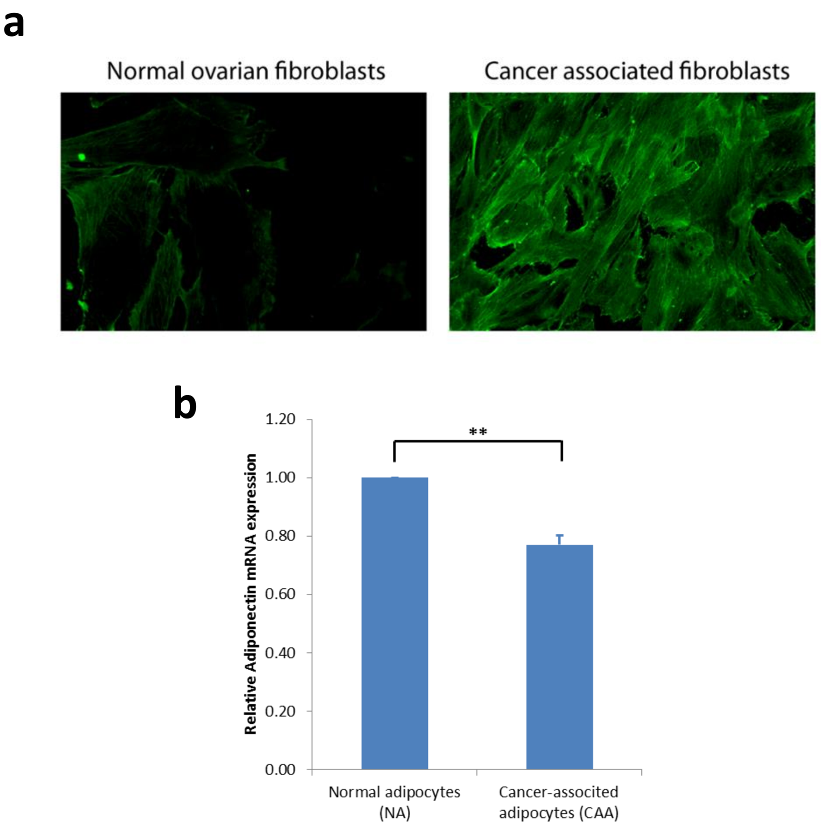

Supplementary Figure 1. Characterization of cancer-associated fibroblasts (CAFs) and cancer-associated adipocytes (CAAs). (**a**) The α-SMA expression levels in normal ovarian fibroblast (n=2) and CAF (n=3) primary cultures were examined using immunofluorescence analysis. Cells on glass slides were fixed with 3.7% formaldehyde, followed by Alexa 647 conjugated phalloidin staining, and mounted using antifade reagent. The stained cells were then observed using confocal microscopy. Representative microscopic images were illustrated. (**b**) The relative adiponectin expression levels in normal adipocytes (n=3) and CAA (n=3) primary cultures were examined using qRT-PCR analysis. Mean ± s.d.; ** $p<0.01$; two-tailed Student's t-test.

Supplementary Figure-1

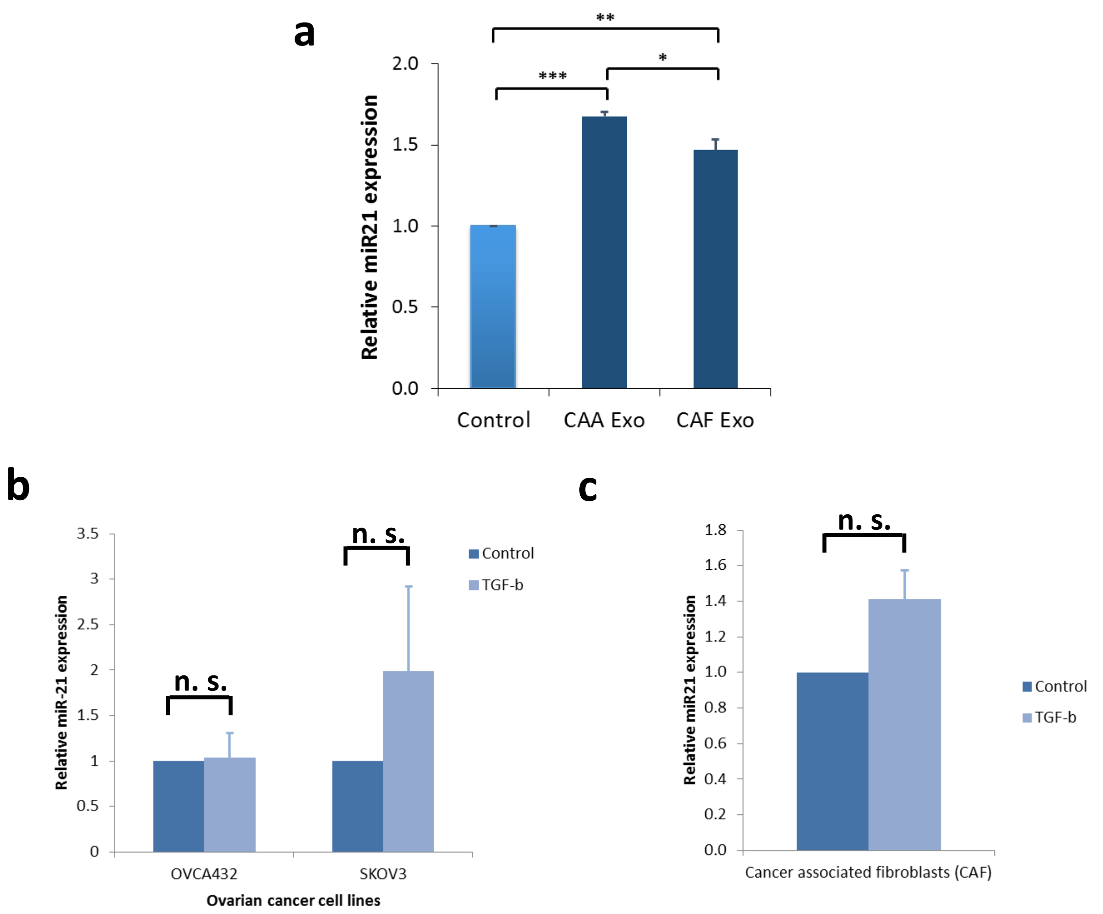

Supplementary Figure 2. Relative miR21 expression levels in ovarian cancer cells and cancer-associated fibroblasts (CAFs). (**a**) Ovarian cancer SKOV3 cells incubated with cancer-associated adipocyte (CAA)- and CAF-derived exosomes showed higher miR21 expression levels than did controls. The results were average from at least three independent experiments. Mean ± s.d.; *** P<0.001, ** p<0.01, * p<0.05; two-tailed Student's t-test. (**b**) OVCA432 and SKOV3 ovarian cancer cells and (**c**) CAFs treated with TGF-β showed no significant change in miR21 expression. The results were the average from at least three independent experiments. Mean ± s.d.; n.s.: not significant (p>0.05; two-tailed Student's t-test).

Supplementary Figure-2

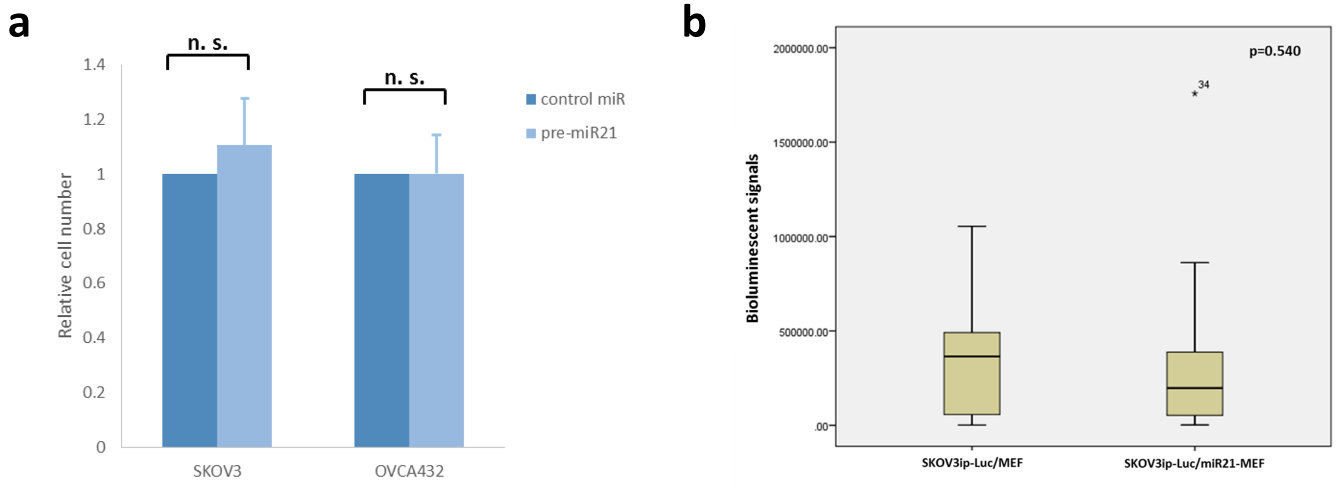

Supplementary Figure 3. The effect of miR21 on cell proliferation *in vitro* and *in vivo*. (**a**) Effect of miR21 overexpression on cell proliferation of SKOV3 and OVCA432 ovarian cancer cells. Cells were incubated for 72 hours after transient transfection with miR21 precursor (pre-miR21) or control miR. Relative cell number was measured by MTT assay. Bar chart showing there is no significant changes in relative cell number with or without miR21 overexpression. The results were averaged from at least three independent experiments. Mean ± s.d.; n.s.: not significant (p>0.05; two-tailed Student's t-test). (**b**) Luciferase-labeled SKOV3ip ovarian cancer cells and $^{miR21+/miR21+}$MEF (miR21-MEF) or $^{miR21-/miR21-}$MEF (MEF) cells were subcutaneously injected into female BALB/c athymic nude mice at the age of 6 weeks to establish tumors. The tumor volumes were measured and quantified using the IVIS-Lumina XR in vivo imaging system 5 days post-injection. A box plot showing no significant changes in luciferase activity between the miR21-MEF group (n=17) and the MEF group (n=17) (p=0.540; Mann-Whitney U test).

Supplementary Figure-3

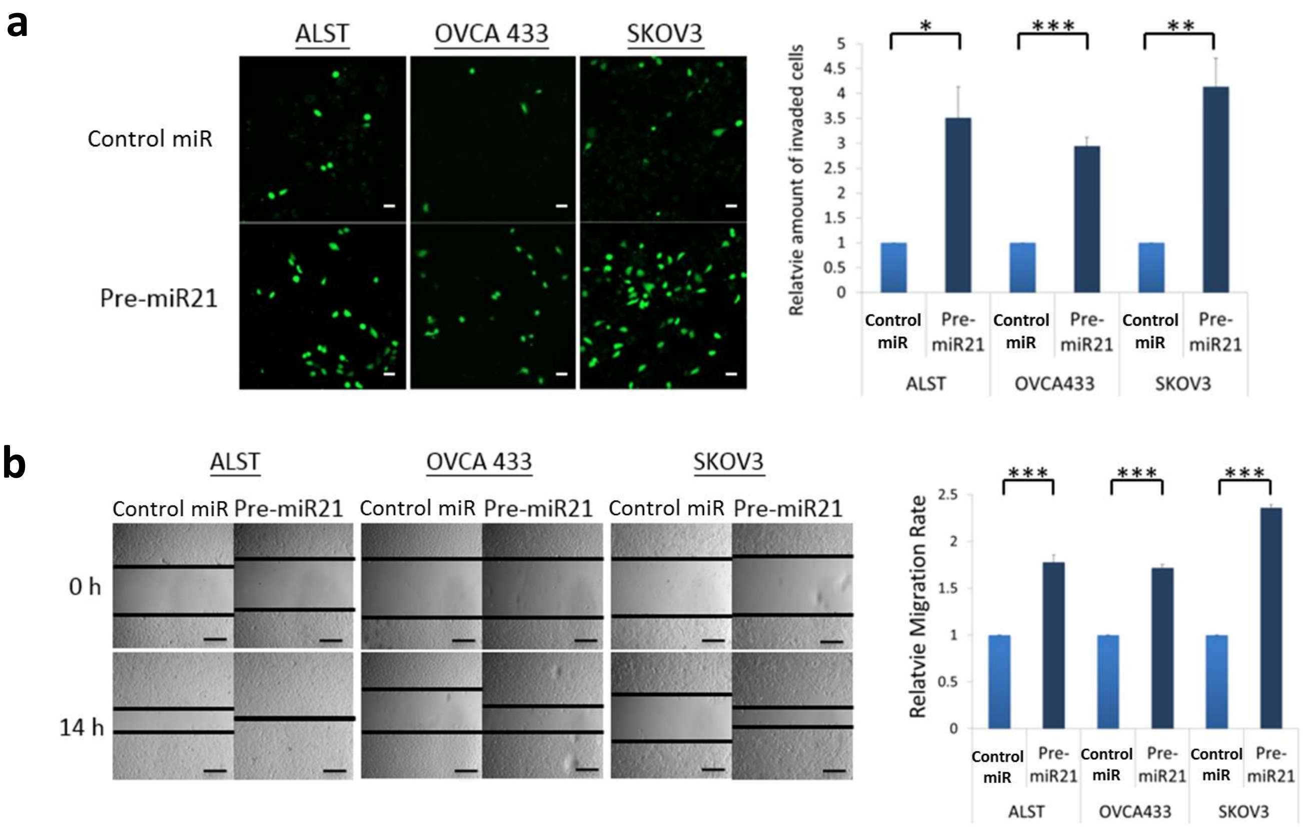

Supplementary Figure 4. Effect of miR21 on ovarian cancer cell invasion potential. (**a**) ALST, OVCA433, and SKOV3 cells were transfected with miR21 precursor (pre-miR21) or negative control (control miR) and seeded onto a transwell plate coated with type I collagen matrix. The cells were allowed to invade for 24 h. Invading cells were then fluorescently labeled with calcein and quantified. Representative microscopic images were illustrated. Bar=10 μm. The results in the bar chart were the average from at least three separate experiments. Mean ± s.d.; *** P<0.001, ** P<0.01, * P<0.05; two-tailed Student's t-test. (**b**) Effect of miR21 on ovarian cancer cell migration. Cells were transfected with miR21 precursor (pre-miR21) and grown to confluence before scratching. The wound closure was photographed at 0 and 14 h. The migration distance of each sample was first normalized to the initial width of the wound and then compared with the control sample. Representative microscopic images were illustrated. Bar=50 μm. The results in the bar chart were the average from at least three separate experiments with duplicated samples. Mean ± s.d.; *** P<0.001; two-tailed Student's t-test.

Supplementary Figure-4

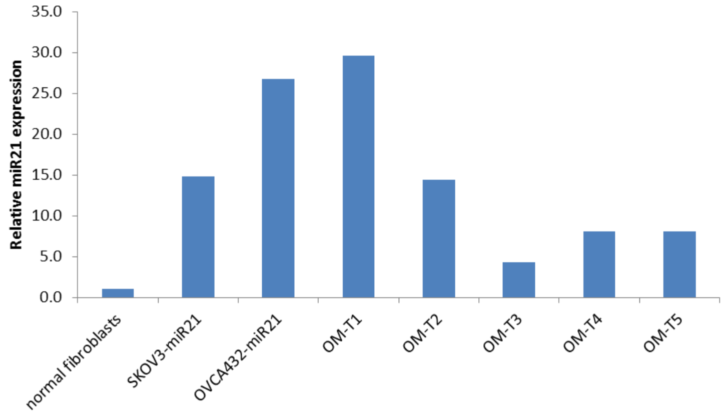

Supplementary Figure 5. The relative miR21 expression between transient miR21 overexpressed ovarian cancer cells and physiological levels. The relative miR21 expression levels in miR21-overexpressing ovarian cancer SKOV3 (SKOV3-miR21) and OVC432 (OVCA432-miR21) cells were examined using quantitative RT-PCR analysis. miR21 expression levels were increased by about 15 to 25 fold compared to in normal fibroblasts. Physiological levels of miR21 were determined in the microdissected epithelial components of malignant ovarian tissues obtained from ovarian cancer patients (OM-T1 to OM-T5). The highest miR21 expression level was about a 30-fold increase, with an average of about a 10-fold increase compared to normal fibroblasts.

Supplementary Figure-5

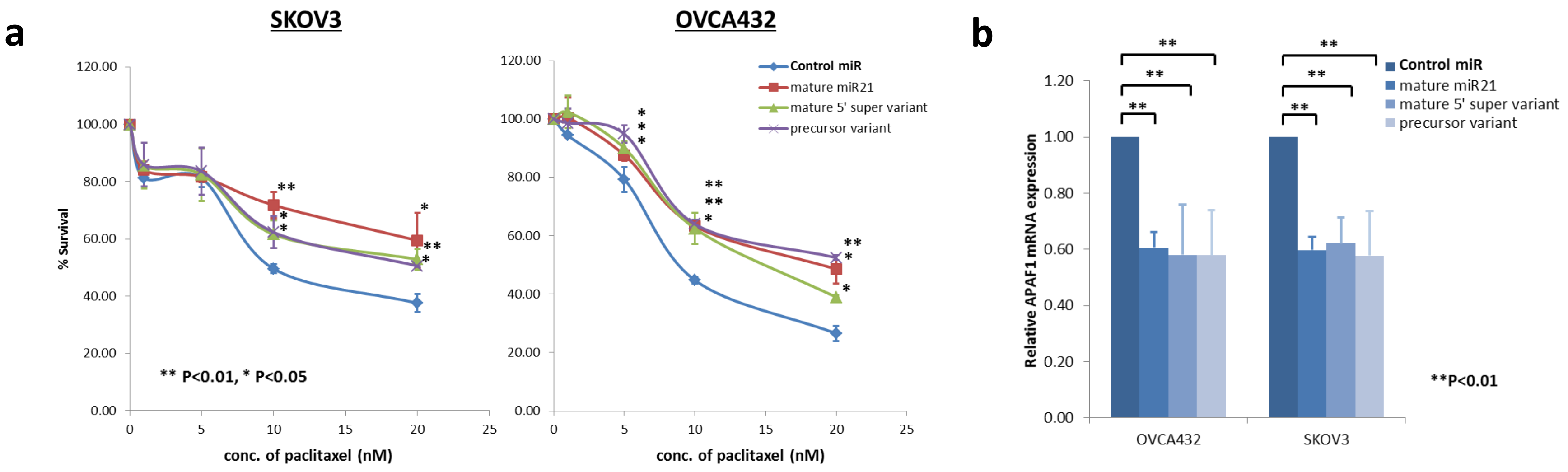

Supplementary Figure 6. Effect of miR21 isomiRs on chemosensitivity in ovarian cancer cells. (**a**) Overexpression of miR21 isomiRs decreased paclitaxel sensitivity in SKOV3 and OVCA432 ovarian cancer cells. After transfection with mature miR21 mimic (mature miR21), isomiR mimics (mature 5’ super variant and precursor variant) or negative control (control miR), the cells were incubated with paclitaxel for 3 days. Cell survival was measured using the MTT assay. The results were the average from at least three independent experiments. Mean ± s.d.; ** p<0.01, * p<0.05; two-tailed Student’s t-test. (**b**) OVCA432 and SKOV3 ovarian cancer cells, transiently transfected with mature miR21 mimic (mature miR21) or isomiR21 mimics (mature 5’ super variant and precursor variant), had a significantly lower level of APAF1 mRNA than did the negative mimic control (control miR). The results were the average from at least three independent experiments. Mean ± s.d.; ** p<0.01; two-tailed Student’s t-test.

Supplementary Figure-6

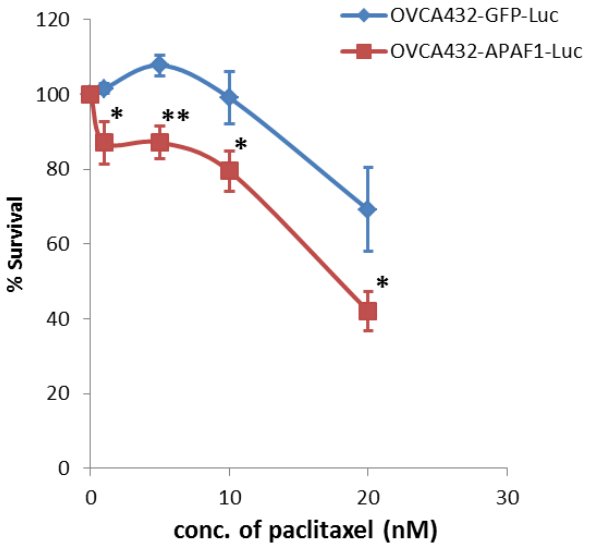

Supplementary Figure 7. Effect of doxycycline-inducible APAF1-stably transfected OVCA432 cells on chemosensitivity *in vitro*. Overexpression of APAF1 by doxycycline induction increased paclitaxel sensitivity in OVCA432 ovarian cancer cells (OVCA432-APAF1-Luc) compared to in control cells (OVCA432-GFP-Luc). The cells were incubated with doxycycline for 48 h before the 3-day paclitaxel treatment. Cell survival was measured by MTT assay. The results are the average of at least three independent experiments. Mean ± s.d.; ** P<0.01, * P<0.05; two-tailed Student's t-test.

Supplementary Figure-7

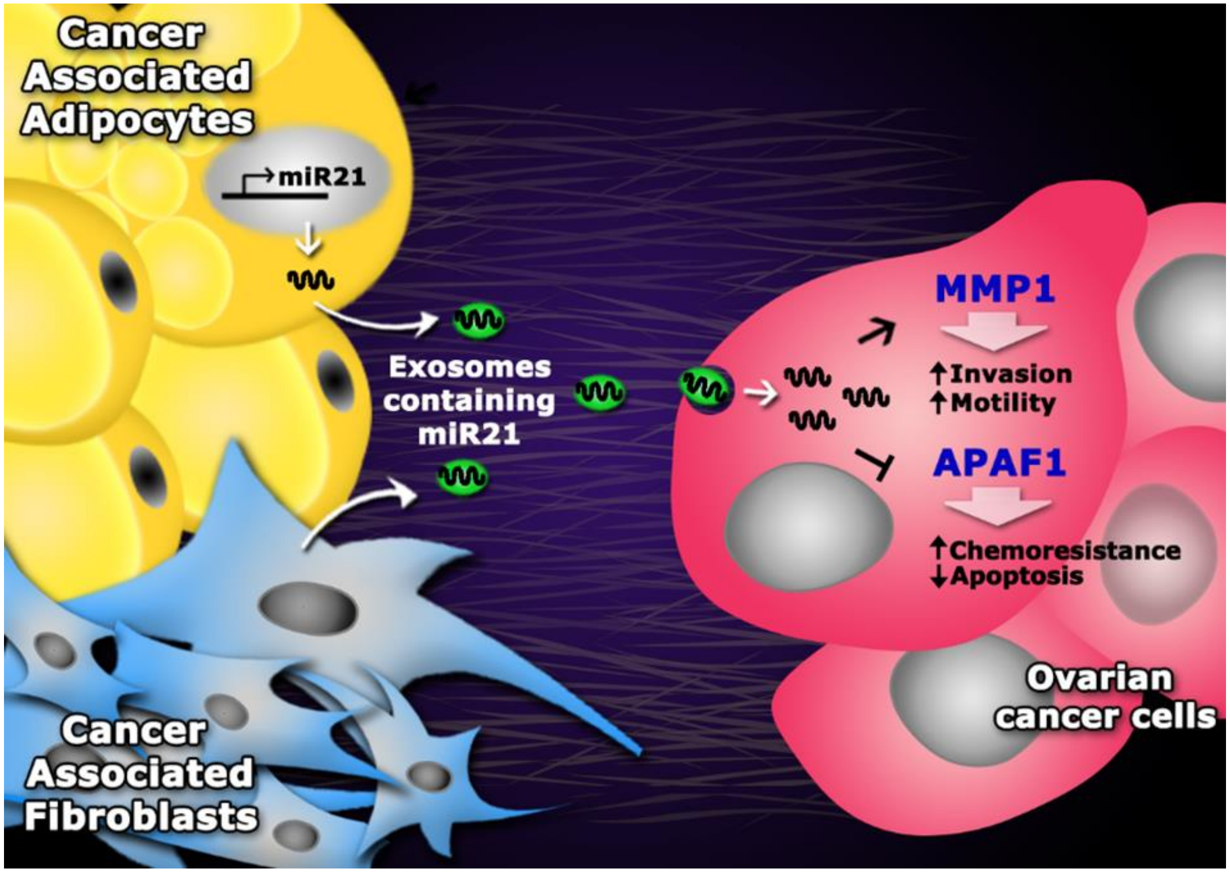

Supplementary Figure 8. Exosomal transfer of stromal cell-derived miR21 in ovarian cancer cell progression. Schematic diagram showing a hypothetical model illustrating that the secretion of miR21 by CAAs and CAFs activates miR21-mediated ovarian cancer cell progression.

Supplementary Figure-8

Figure 1c.

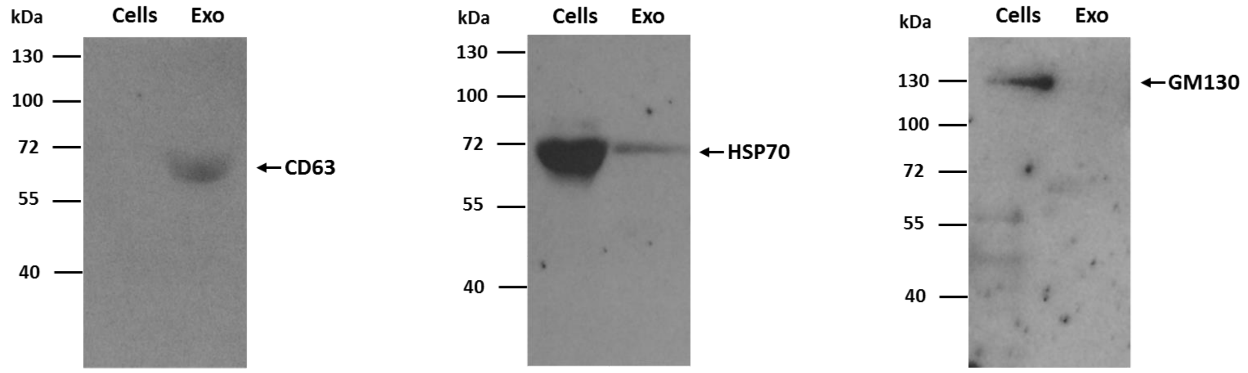

Supplementary Figure 9. Original images of blots for Figure 1c.

Supplementary Figure-9

Figure 5f.

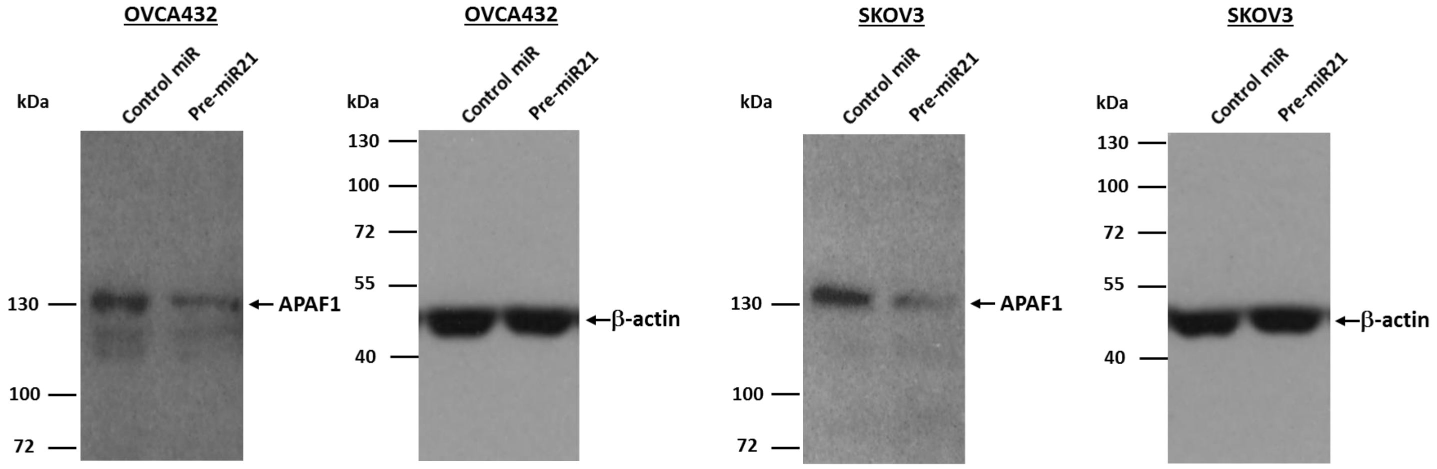

Supplementary Figure 10. Original images of blots for Figure 5f.

Supplementary Figure-10

Supplementary Table 1. Concentration of exosomes isolated from different cell types

Cell types	Volume of conditioned medium collected (mL)	Concentration of exosomes (particles/mL)
Cancer associated adipocytes (CAA) (n=5)	20	$4.8x10^{8} – 7.9x10^{8}$
Cancer associated fibroblasts (CAF) (n=3)	24	$1.2x10^{10} – 3.9x10^{11}$
Ovarian cancer SKOV3 cells (n=2)	24	$1.1x10^{9}$

Supplementary Table 2. Relative expression of different miR21 variants measured using miRNA-sequencing and quantitative RT-PCR analyses

	**mature miR21**	relative miRNA expression	
	total copy number per 1000 cells	miRNA-sequencing	qRT-PCR
OVCA (n=4)	498	1	1
NF (n=2)	1294	2.598393574	2.444111644
CAF (n=3)	1317	**2.644578313**	**2.152546227**
NA (n=2)	140064	281.253012	44.74219594
CAA (n=2)	2751956	**5526.016064**	**3266.849175**

	**mature 5' super variant**	relative miRNA expression	
	total copy number per 1000 cells	miRNA-sequencing	qRT-PCR
OVCA (n=4)	3339	1	1
NF (n=2)	17692	5.298592393	2.98742402
CAF (n=3)	10168	**3.045223121**	**1.40141971**
NA (n=2)	496782	148.7816712	39.37544502
CAA (n=2)	6759635	**2024.448937**	**2928.319547**

	**precursor variant**	relative miRNA expression	
	total copy number per 1000 cells	miRNA-sequencing	qRT-PCR
OVCA (n=4)	42	1	1
NF (n=2)	481	11.45238095	3.419696946
CAF (n=3)	215	**5.119047619**	**2.789310745**
NA (n=2)	7549	179.7380952	39.93148833
CAA (n=2)	58744	**1398.666667**	**2209.677602**

Supplementary Table 3. Mean fold changes of top ten genes in miR21 transfected SKOV3 cells versus the mock transfectants

Gene function	AffID	Gene.Symbol	mean.control	mean.miR21	FoldChange.miR21.vs.control	Raw.Pvalues
Apoptosis	201466_s_at	JUN	6.94	5.66	-2.43	0.046450188
	208796_s_at	CCNG1	10.7	9.59	-2.16	0.024181327
	209970_x_at	CASP1	5.37	4.41	-1.94	0.032098575
	204859_s_at	APAF1	3.77	2.82	-1.93	0.021815295
	212185_x_at	MT2A	12.83	12.09	-1.66	0.026881785
	222985_at	YWHAG	10.89	11.63	1.67	0.007132634
	208946_s_at	BECN1	8.82	9.56	1.67	0.019541405
	226048_at	MAPK8	9.14	9.91	1.71	0.002705789
	206075_s_at	CSNK2A1	7.43	8.31	1.84	0.028234387
	204604_at	PFTK1	8.37	9.37	2	0.016226057
Metastasis	210253_at	HTATIP2	7.73	6.32	-2.67	0.046076182
	202628_s_at	SERPINE1	7.63	6.24	-2.62	0.043072609
	213139_at	SNAI2	7.92	6.77	-2.22	0.033949968
	210665_at	TFPI	4.49	5.43	1.92	0.001371032
	202011_at	TJP1	9.59	10.54	1.93	0.013713295
	221911_at	ETV1	2.62	3.7	2.11	0.042054977
	212397_at	RDX	9.94	11.16	2.32	0.043734302
	211478_s_at	DPP4	7.31	8.55	2.36	0.01786473
	227539_at	GNA13	4.75	6.42	3.18	0.007467805
	204475_at	MMP1	8.12	9.93	3.52	0.038152613
